# Kinetics and prognostic value of heparin binding protein at the ST-segment-elevation myocardial infarction

**DOI:** 10.1080/07853890.2026.2622182

**Published:** 2026-01-30

**Authors:** Yueying Wang, Tianqi Zhu, Wenli Zhang, Ruiyan Zhang, Hongyang Xie, Xuezheng Qu, Keremu Buayiximu, Zhengbin Zhu, Jingwei Ni, Run Du, Jinzhou Zhu, Xiaoqun Wang, Fenghua Ding, Xiaoxiang Yan, Ping Li, Zhihong Xu, Weiwei Quan

**Affiliations:** a Department of Cardiovascular Medicine, Ruijin Hospital, Shanghai Jiao Tong University School of Medicine, Shanghai, China; b Institute of Cardiovascular Diseases, Shanghai Jiao Tong University School of Medicine, Shanghai, China; c Department of Geriatrics, Ruijin Hospital, Shanghai Jiaotong University School of Medicine, Shanghai, China

**Keywords:** Heparin binding protein, acute ST-segment elevation myocardial infarction, heart failure, major adverse cardiac events

## Abstract

**Background:**

This research aimed to explore the potential role of heparin-binding protein (HBP) as a prognostic biomarker for adverse events among individuals diagnosed with ST-segment elevation myocardial infarction (STEMI) who underwent primary percutaneous coronary intervention (pPCI).

**Methods:**

This cohort study enrolled 215 consecutive patients with STEMI following pPCI. Plasma HBP and high-sensitivity cardiac troponin I (hs-cTnI) levels were measured at admission, and at 24h, 48h, and 72h after pPCI. During a median follow-up period of 1.5 years, major adverse cardiovascular events (MACE) were recorded.

**Results:**

Plasma HBP levels decreased from a median of 58.04 (IQR 30.38, 106.04) ng/mL at admission to 23.80 (IQR 14.03, 44.51) ng/mL at 72 h (HBP_72h_) after pPCI (*p* < 0.001). HBP levels demonstrated a moderate correlation with hs-cTnI, particularly at 72 h post-pPCI (*r* = 0.56). Multivariable Cox regression analysis demonstrated that the highest HBP_72h_ quartile was independently associated with a 5-fold increased risk of MACE during follow-up compared to the lowest quartile (hazard ratio, 5.156; 95% confidence interval, 1.380, 19.271; *p* = 0.0148). The ROC curve demonstrated that an HBP_72h_ cut-off level of 29.12 ng/mL for predicting MACE had a specificity of 78.0% and a sensitivity of 61.9%, with an AUC of 0.730. Furthermore, adding HBP_72h_ to hs-cTnI improved MACE prediction compared to hs-cTnI alone (NRI 0.578; *p* < 0.001).

**Conclusions:**

Elevated HBP levels following STEMI were associated with an increased risk of adverse outcomes and may serve as a novel and valuable prognostic marker in patients with STEMI.

## Graphical Abstract

**Figure:**
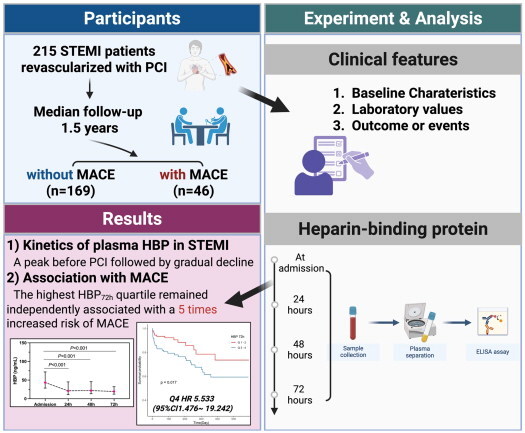

## Introduction

Despite advances in treatment, individuals with acute myocardial infarction (AMI) remain at substantial risk of cardiovascular complications [1]. Current guidelines highlight the importance of prompt risk evaluation following AMI to guide appropriate therapeutic interventions. Accurate risk stratification is essential for recognizing high-risk patients, guiding clinical decision-making, and ultimately improving outcomes [2]. Therefore, there is a pressing need to identify new and reliable biomarkers that can predict poor outcomes in patients with AMI, beyond the traditionally recognized risk factors.

Heparin-binding protein (HBP), also known as CAP37/azurocidin, is a member of the serprocidin family of neutrophil-derived cationic proteins. It is pre-synthesized and stored in neutrophil secretory vesicles and rapidly released into circulation upon neutrophil activation, leading to a sharp rise in plasma concentrations. In addition to its neutrophil origin, HBP plays a critical role in endothelial barrier disruption and inflammatory responses [3]. Previous research has explored HBP as a promising biomarker in sepsis, demonstrating its superior predictive value over traditional markers such as C-reactive protein (CRP), white blood cell (WBC) count, procalcitonin, and lactate in assessing disease severity and progression [4,5]. Moreover, a recent study revealed that HBP is released from the reperfused coronary circulation, along with neutrophil adhesion and myocardial injury, suggesting its potential role in ischemia-reperfusion processes [6]. However, the association between HBP and clinical outcomes in patients with ST-segment elevation myocardial infarction (STEMI) remains poorly understood.

Given these findings, we aimed to determine whether HBP could serve as a novel prognostic biomarker for adverse outcomes in patients with STEMI following primary percutaneous coronary intervention (pPCI). To address the knowledge gap, we conducted a prospective cohort study to evaluate the hypothesis that elevated HBP levels are associated with poor clinical outcomes in patients with STEMI.

## Methods

### Study population and ethics approval

This was a prospective, observational cohort study that evaluated plasma HBP levels in patients with STEMI. The study population was derived from the Ruijin Acute ST-Segment Elevation Myocardial Infarction (RJH-STEMI, NCT05450757) cohort, a prospective registry conducted at Ruijin Hospital, Shanghai Jiao Tong University School of Medicine.

From July 2022 to June 2023, we enrolled 251 consecutive patients with STEMI who underwent pPCI at Ruijin Hospital. Each patient had accepted guideline-directed medical therapy during their index STEMI hospitalization [2,7]. STEMI diagnosis was based on the Fourth Universal Definition of Myocardial Infarction [8]. Exclusion criteria included: (1) pre-existing cardiovascular diseases, including coronary artery disease, prior myocardial infarction (MI), coronary revascularization, heart failure (HF), or severe valvular disease; (2) systemic comorbidities, including renal failure, autoimmune disorders, chronic inflammatory conditions, malignancies, or hematologic diseases; (3) active/chronic infections; and (4) current use of medications known to significantly affect HBP levels, such as systemic corticosteroids, immunosuppressants, or non-steroidal anti-inflammatory drugs. The detailed inclusion/exclusion criteria are presented in Supplementary Figure 1.

This study was approved by the Institutional Research Ethics Committee of Ruijin Hospital in Shanghai Jiao Tong University School of Medicine (Approval No. 2020-152) and was approved by the Declaration of Helsinki (revised). All participants or their legal guardians provided written informed consent for data collection and blood sampling.

### Blood sampling and measurements

Blood samples were collected at four time points: at admission (HBP_0h_, during coronary angiography) and at 24 h (HBP_24h_), 48 h (HBP_48h_), and 72 h (HBP_72h_) after admission (Supplementary Figure 2). At each time point, 5 mL of EDTA-anticoagulated blood was obtained *via* aseptic venipuncture from the antecubital vein and centrifuged at 2,200 g for 15 min. All samples were processed within 2 h of collection. A Jet-iStar 3000 automatic analyzer (JOINSTAR BIOMEDICAL TECHNOLOGY CO., LTD, Hangzhou, China) was used to analyze 50 µL of sample, which was incubated for 18 min, and HBP levels were measured using a dry fluorescent immunoassay. This assay is based on a double-antibody sandwich immunofluorescence principle. Briefly, the sample reacts with fluorescent microspheres conjugated to anti-HBP monoclonal antibodies, and the resulting fluorescence intensity is directly proportional to the HBP concentration. According to the manufacturer’s instructions, the lower limit of detection for this assay is 5.90 ng/mL, with a linear measurement range of 5.90–300.00 ng/mL. All assays were performed by technicians blinded to clinical data and patient identity.

**Figure 2.**
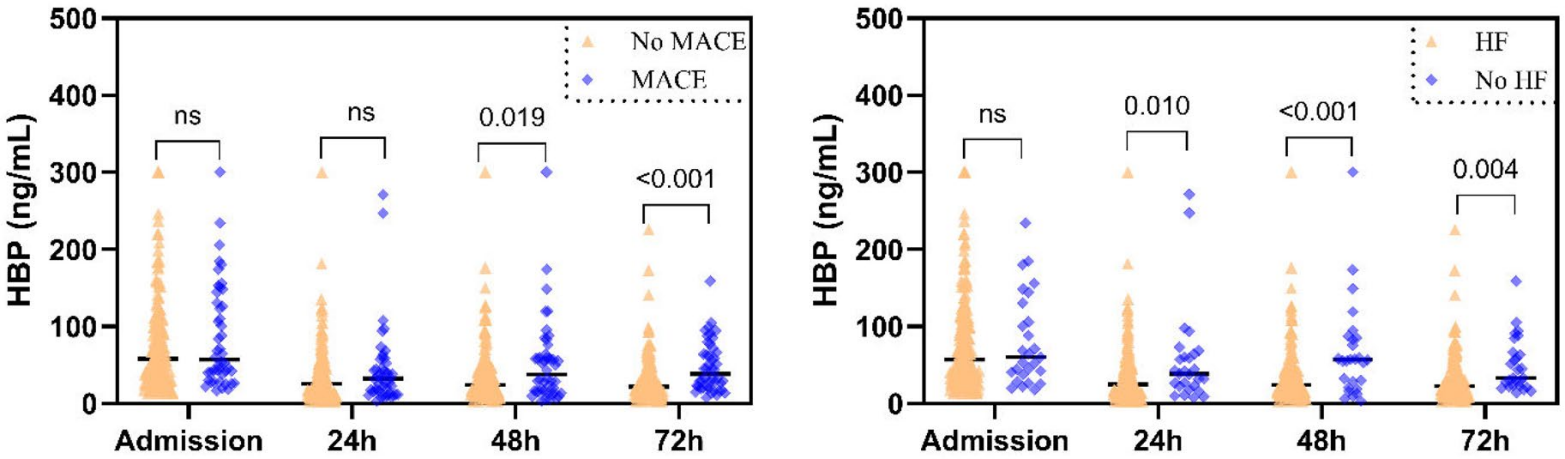
Distribution of HBP levels over time according to clinical outcomes. (A) Plasma HBP levels according to MACE (all-cause mortality, hospitalization for heart failure, relapse MI, or stroke); (B) plasma HBP levels according to hospitalization for heart failure. Wilcoxon matched-pairs signed rank test was used to compare HBP levels at different time points. HBP: heparin-binding protein; MACE: major adverse cardiac events; HF: heart failure.

### Data collection

All demographic and clinical characteristics were collected through face-to-face interviews at admission, which was set as the baseline. Echocardiography was performed at 1 day and 180 ± 15 days after percutaneous coronary intervention (PCI). Furthermore, regular laboratory tests, including levels of glucose and lipids, liver and renal function, blood routine examination, and interferon (IFN)α, IFNγ, tumor necrosis factor (TNF)α, interleukin (IL)-1β, IL-2, IL-4, IL-5, IL-6, IL-8, IL-10, IL-17, and IL-20p70 were measured at the 1st day after PCI as the inflammatory markers. High sensitivity CRP (hs-CRP), creatine kinase (CK), creatine kinase myocardial band (CK-MB), and high-sensitivity Troponin I (hs-TnI) and were analyzed at the same time points as HBP. Data were collected on therapy at discharge, including beta-blockers, angiotensin-converting enzyme inhibitors (ACEIs)/angiotensin receptor blockers (ARBs)/angiotensin receptor-neprilysin inhibitors (ARNIs), Statins, and mineralocorticoid receptor antagonists (MRAs). The estimated glomerular filtration rate (eGFR) was calculated using the Chronic Kidney Disease Epidemiology Collaboration equation [9]. Body mass index (BMI) (kg/m^2^) was calculated using height and weight measured at baseline. The definitions of demographic and clinical characteristics were shown in the Supplementary Table 1. The handling of missing values was performed using the multiple imputation method, detailing proportion of missing data described in the Supplementary Table 2.

### Echocardiography

Echocardiography was performed by a cardiologist with experience in advanced echocardiography and trained for the requirements of the study, using standard parasternal and apical views with frame rates of 45–75 frames/s, with a GE Vivid E9 ultrasound system (GE Vingmed Ultrsound, Horton, Norway) equipped with a 1.7–3.4 MHz transducer and following current recommendations for cardiac chamber quantification in adults [10–12]. Echocardiography was performed by the same cardiologist, who was blinded to the clinical data and electrocardiographic data.

### Study endpoints

All patients were followed up at scheduled intervals: 30 ± 7 days, 180 ± 15 days, 365 ± 30 days, and every 6 months after the last visit. To identify the main outcome, follow-up was carried out prospectively by phone, in-person interviews, and chart reviews. Major adverse cardiac events (MACE) were the main endpoint; this was defined as a composite of all-cause mortality, myocardial infarction, stroke or hospitalization for unstable angina or HF. Based on the definitions of the Cardiovascular Trials Initiative for Standardized Data Collection, all endpoints were adjudicated [13]. The detailed definitions of endpoints and their components are available in the Definition of study endpoints section. An endpoint adjudication committee, which was blinded to the plasma HBP data, reviewed source documents to confirm events and assess the need for hospitalization. The follow-up period was computed using the date of death or the last recorded healthcare encounter.

### Statistical analysis

Statistical analyses were performed using R version 3.5.1 (R Studio, Boston, MA) and GraphPad Prism version 9.5.1 (GraphPad Software, San Diego, CA, USA). All tests were two-sided, and a *P-*value < 0.05 was considered statistically significant.

Depending on the data distribution, which was evaluated using the Kolmogorov-Smirnov test, continuous variables are expressed as mean standard deviation (SD) or median (interquartile range [IQR]). Categorical variables are presented as numbers (percentages).

For HBP release kinetics, we applied for the Friedman test, followed by Nemenyi post-hoc analysis. Mixed-effects models were used for between-group comparisons. Continuous variables were compared between the MACE and non-MACE groups using Student’s t-test or the Wilcoxon rank-sum test. Differences across HBP quartiles were assessed using one-way ANOVA or the Kruskal–Wallis test, as appropriate. Pearson’s correlation analysis was performed on log10-transformed HBP values and inflammatory markers [high-sensitivity CRP (hs-CRP)], as well as myocardial injury markers [creatine kinase (CK), creatine kinase myocardial band (CK-MB), and high-sensitivity cardiac troponin I (hs-cTnI)] at admission, and at 24, 48, and 72 h after pPCI. Missing data for covariates were dealt with using multiple imputations by chained equations, detail proportion of missing data were described in Supplementary Table 2.

For outcome analyses, follow-up continued until the occurrence of a primary endpoint or the last documented visit, whichever occurred first. The association between baseline HBP levels and MACE risk was assessed using Cox proportional hazards models, with results presented as hazard ratios (HRs) and 95% confidence intervals (CIs). Covariates included in the multivariable Cox regression models were selected a priori based on clinical plausibility and established prognostic relevance in patients with STEMI. These covariates encompassed demographic characteristics, traditional myocardial risk factors, and key hemodynamic and laboratory parameters consistent with validated risk stratification tools such as the GRACE score [14]. The following three sequential models were constructed:

1. Unadjusted Cox model.
2. Partially adjusted model incorporating age and sex (female [referent], male).
3. Fully adjusted model including age, sex, body mass index (BMI), smoking status (no [referent], yes), baseline systolic blood pressure (SBP), hypertension (no [referent], yes), diabetes mellitus (no [referent], yes), Killip classification (class I [referent], classes II–IV), WBC count, estimated glomerular filtration rate (eGFR), peak hs-CRP, and peak hs-cTnI.

The proportional hazards assumption for all the Cox regression models was evaluated using scaled Schoenfeld residuals, and global tests indicated no violations of this assumption (all *p* > 0.05). Multicollinearity among covariates was assessed using the variance inflation factor (VIF); all VIF values were < 2, indicating no significant collinearity. Event-free survival was assessed using Kaplan-Meier analysis, with between-group comparisons performed using the log-rank test. Additionally, we used landmark analysis to assess the impact of HBP levels during the 180-day landmark period on the composite endpoint. Receiver operating characteristic (ROC) curves were generated to evaluate predictive performance, and areas under the ROC curves (AUCs) with 95% CIs were reported. The Youden Index was used to derive optimal cut-off values from the ROC curves, and sensitivities and specificities were calculated for these cut-off values with 95% CIs. Harrell’s C-statistic was computed from the Cox proportional hazards regression model [15]. Model performance and discrimination ability were further evaluated using C-statistics and the net reclassification index (NRI) [16]. Statistical power was assessed based on the number of observed events required for Cox proportional hazards regression. According to the Schoenfeld formula, approximately 38 outcome events were required to detect a HR of 2.5 for the highest versus the lowest quartile with 80% power at a two-sided significance level of 0.05. In this study, the enrollment of 215 patients resulted in 46 MACE events (21.4%) during follow-up, providing sufficient statistical power (>80%) to validate the prognostic value of HBP.

Linear regression was used to assess the relationship between HBP levels and HF, with left ventricular ejection fraction (LVEF) at 1 day and at 180 ± 15 days post-pPCI as continuous dependent variables. Moreover, Fine-Gray competing risk regression was employed to examine the association between HBP levels and HF events, with all-cause mortality treated as a competing risk.

## Results

### Study population and baseline characteristics

Table 1 presents the baseline characteristics of 215 patients with STEMI stratified by MACE occurrence. Compared to the non-MACE group (*n* = 169), patients in the MACE group (*n* = 46) were older (68.3 ± 12.0 vs. 63.1 ± 12.1 years, *p* = 0.011) and had lower SBP (114 [IQR 102,143] vs. 127 [IQR 110,146] mmHg, *p* = 0.046). Moreover, the MACE group exhibited higher rates of chronic kidney disease (15.2% vs. 4.7%, *p* = 0.014) and Killip class III–IV (15.2% vs. 3.6%, *p* < 0.001). Medication analysis revealed that patients in the MACE group were more frequently prescribed mineralocorticoid receptor antagonists (28.3% vs. 6.5%, *p* < 0.001). Additionally, at baseline, patients in the MACE group had larger cardiac dimensions (left ventricular end-diastolic diameter [LVEDD]: 50.98 ± 5.90 vs. 49.55 ± 4.65 mm; *p* = 0.03) and reduced LVEF (51.98 ± 9.57% vs. 58.70 ± 7.42%, *p* < 0.01).

**Table 1.** Clinical characteristics of patients at baseline.

	Total (*n* = 215)	non-MACE (*n* = 169)	MACE (*n* = 46)	*P* value
Demographic and risk factors				
Age, year	64.17 ± 12.26	63.05 ± 12.14	68.28 ± 11.95	0.011
Male, n (%)	170 (79.07)	136 (80.47)	34 (73.91)	0.332
Current smoking, n (%)	51 (23.72)	40 (23.67)	11 (23.91)	0.517
BMI, kg/m^2^	24.95 ± 3.65	25.07 ± 3.61	24.49 ± 3.79	0.361
Admission heart rate, bpm	82 (74, 94)	82 (74, 92)	84 (77, 105)	0.036
Admission SBP, mmHg	126 (109, 144)	127 (110, 146)	114 (102, 143)	0.046
Admission DBP, mmHg	77 (67, 87)	78 (69, 88)	74 (66, 81)	0.067
Comorbidities				
Diabetes mellitus, n (%)	60 (27.91)	42 (24.85)	18 (39.13)	0.056
Hypertension, n (%)	147 (68.37)	115 (68.05)	32 (69.57)	0.844
Chronic kidney disease, n (%)	15 (6.98)	8 (4.73)	7 (15.22)	0.014
Killip classification, n (%)				<0.001
I	186 (86.5)	159 (94.1)	27 (58.7)	
II	16 (7.4)	11 (6.5)	5 (10.9)	
III-IV	13 (6.0)	6 (3.6)	7 (15.2)	
Culprit lesion, n (%)				0.675
LAD	105 (48.8)	79 (46.7)	26 (56.5)	
LCX	43 (20.0)	37 (21.9)	6 (13.0)	
RCA	67 (31.2)	52 (30.8)	15 (32.6)	
Stent length, mm	26.63 ± 6.32	26.32 ± 6.43	27.97 ± 5.70	0.208
Stent diameter, mm^2^	3.00 ± 0.39	3.01 ± 0.40	2.97 ± 0.31	0.544
Non-culprit lesions, n (%)	146 (67.9)	117 (69.2)	29 (63.0)	0.426
Laboratory values				
WBC, ×10^9^/L	10.60 (8.76, 12.86)	10.25 (8.75, 12.19)	12.22 (8.83, 15.68)	0.004
Hemoglobin, g/L	138 (125, 150)	139 (130, 150)	126 (111, 146)	0.003
Platelet, ×10^9^/L	214 (177, 254)	212 (175, 248)	224 (187, 270)	0.081
Fasting glucose, mmol/L	6.65 (5.70, 8.74)	6.59 (5.66, 7.84)	8.32 (6.19, 12.79)	0.006
HbA1c, %	6.00 (5.60, 7.00)	5.90 (5.60, 6.80)	6.00 (5.70, 8.10)	0.145
Creatine, μmol/L	78 (67, 96)	76 (66, 88)	91 (73, 119)	0.018
eGFR, mL/minute/1.73m^2^	84.80 (67.90, 96.50)	88.10 (73.80, 98.70)	69.30 (57.00, 80.20)	<0.001
Triglyceride, mmol/L	1.62 (1.13, 2.26)	1.52 (1.05, 2.01)	1.71 (1.15, 2.33)	0.152
Total cholesterol, mmol/L	4.93 (4.05, 5.64)	4.52 (3.62, 5.48)	4.95 (4.29, 5.70)	0.043
HDL-C, mmol/L	1.03 (0.89, 1.22)	1.03 (0.88, 1.21)	1.03 (0.91, 1.22)	0.690
LDL-C, mmol/L	3.03 (2.51, 3.64)	2.74 (1.95, 3.50)	3.08 (2.53, 3.69)	0.048
Lp(a), mmol/L	0.15 (0.07, 0.37)	0.13 (0.07, 0.32)	0.31 (0.11, 0.48)	0.003
Peak NT-proBNP, pg/ml	1191.00 (497.70, 3115.00)	824.50 (445.20, 2012.00)	3648.00 (1560.00, 7502.00)	0.001
Peak hs-CRP, mg/L	70.77 (22.07, 160.15)	43.02 (17.45, 140.17)	153.22 (107.40, 228.53)	<0.001
Peak CK, IU/L	1084.0 (436.00, 1844.0)	989.00 (442.00, 1745.00)	1594.0 (436.00, 2469.00)	0.191
Peak CK-MB, ng/mL	64.20 (17.50, 130.80)	59.50 (24.00, 128.80)	105.95 (10.20, 144.80)	0.655
Peak hs-cTnI, pg/mL	25989.0 (9588.3, 54320.0)	24202.0 (9536.1, 50494.0)	44681.0 (10483.0, 80825.0)	0.047
Medication				
DAPT, n (%)	215 (100)	169 (100)	46 (100)	1.000
ACEIs/ARBs/ARNI, n (%)	161 (74.88)	129 (76.33)	32 (69.57)	0.358
β-blocker, n (%)	153 (71.16)	117 (69.23)	36 (78.26)	0.236
Statins, n (%)	200 (93.02)	160 (94.67)	40 (86.96)	0.071
MRAs, n (%)	24 (11.16)	11 (6.51)	13 (28.26)	<0.001
Echocardiography				
LA, mm	38.92 ± 4.24	38.60 ± 3.99	40.12 ± 4.90	0.03
LVEDD, mm	49.86 ± 4.96	49.55 ± 4.65	50.98 ± 5.90	0.03
LVESD, mm	50.57 ± 5.24	50.18 ± 4.96	52.00 ± 6.01	<0.01
LVEDV, mL	120.34 ± 28.27	118.49 ± 26.33	127.14 ± 33.92	0.03
LVESV, mL	48 (39, 61)	45 (38, 56)	67 (43, 77)	<0.01
LVEF, %	57.26 ± 8.37	58.70 ± 7.42	51.98 ± 9.57	<0.01

Values are mean ± SD, n (%), or median (first quartile, third quartile).

MACE: major adverse cardiac events; BMI: body mass index; HR: heart rate; SBP: systolic blood pressure; DBP: diastolic blood pressure; LAD: left anterior descending; LCX: left circumflex coronary; RCA: right coronary artery; WBC: white blood cell; HbA1c: glycated hemoglobin; eGFR: estimated glomerular filtration; NT-proBNP: N-terminal pro-brain natriuretic peptide; LDL-C: low-density lipoprotein cholesterol; HDL-C: high-density lipoprotein cholesterol; Lp(a): lipoprotein(a); hs-CRP: high sensitivity C reactive protein; CK: creatine kinase; CK-MB: creatine kinase-MB isoenzyme; hs-cTnI: high sensitivity cardiac troponin I; DAPT: dual anti platelet therapy; ACEIs: angiotensin converting enzyme inhibitors; ARBs: angiotensin receptor blockers; ARNI: angiotensin receptor/neprilysin inhibitor; MRAs: mineralocorticoid receptor antagonists; LA: left atrial diameter; LVEDD: left ventricular end-diastolic diameter; LVESD: left ventricular end-systolic diameter; LVEDV: left ventricular end-diastolic volume; LVESV: left ventricular end-systolic volume; LVEF: left ventricular ejection fraction.

### Kinetics

**Figure 1.**
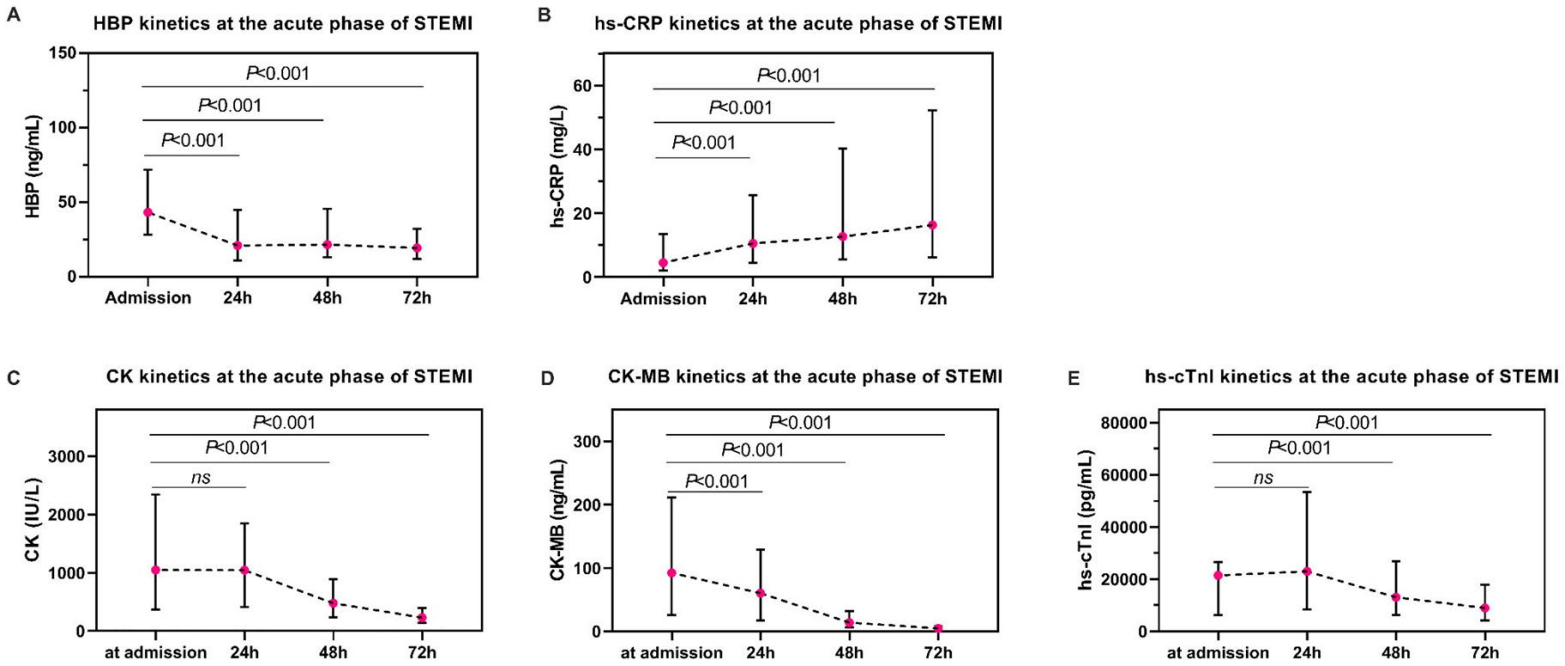
The kinetic profile of HBP, hs-CRP, CK, CK-MB and hs-cTnI during the first 72 h post coronary angiography. Shown for levels of: (A) HBP, (B) hs-CRP, (C) CK, (D) CK-MB, and (E) hs-cTnI release kinetics in a cohort of patients with (STEMI). Round points denote the median level of the corresponding biomarker at the time measured; admission, 24, 48, and 72 h. Solid line represents interquartile range. STEMI: ST-segment elevation myocardial infarction; HBP: heparin-binding protein; hs-CRP: high-sensitive C-reactive protein; CK: creatinine kinase; CK-MB: creatine kinase myocardial band; hs-cTnI: high-sensitive Troponin I.

HBP levels peaked early in STEMI and progressively declined between 24 and 72 h post-pPCI. Plasma HBP levels were a median of 43.43 (IQR 28.60, 71.96) ng/mL at admission, and then gradually decreased to 21.24 (IQR 11.10, 45.21) ng/mL at 24 h, 21.86 (IQR 13.49, 45.84) ng/mL at 48 h, and 19.54 (IQR 12.34, 32.28) ng/mL at 72 h after pPCI (Friedman test, *p* < 0.001; Figure 1A), this is in contrast to hs-CRP (Figure 1B). This kinetic pattern paralleled that observed for myocardial injury markers, such as CK, CK-MB, and hs-cTnI (Figure 1C–E). CK levels remained elevated from a median of 1045.00 (IQR 367.00, 2339.25) IU/L at admission to 24 h [1044.16 (IQR 413.00, 1844.00) IU/L], before declining to 476.00 (IQR 232.00, 889.00) IU/L at 48 h and 229.00 (IQR 137.00, 393.61) IU/L at 72 h. In contrast, CK-MB and hs-cTnI levels peaked at admission and showed a continuous decline. Specifically, CK-MB decreased from a median of 92.45 (IQR 25.85, 211.50) ng/mL at admission to 60.30 (IQR 17.50, 128.80) ng/mL at 24 h, 13.80 (IQR 6.50, 31.90) ng/mL at 48 h, and 4.50 (IQR 2.70, 8.00) ng/mL by 72 h. Similarly, hs-cTnI levels declined from a median of 26586.00 (IQR 21447.45, 68145.20) pg/mL at admission to 24201.80 (IQR 8538.20, 53352.80) pg/mL at 24 h, 13050.40 (IQR 6365.10, 27988.00) pg/mL at 48 h, and 9232.00 (IQR 4250.10, 18137.00) pg/mL by 72 h. Temporal changes for all markers were statistically significant (Friedman test, all *p* < 0.001).

The HBP levels showed no correlation with sex (mixed-effect model, *p* = 0.858), history of hypertension (mixed-effect model, *p* = 0.202), diabetes mellitus (mixed-effect model, *p* = 0.424), LVEF (mixed-effect model, *p* = 0.345), and Killip grade at admission (class 1 or ≥ 1; mixed-effect model, *p* = 0.487) (Supplementary Figure 3).

### Plasma HBP levels in relation to myocardial function

At 180 ± 15 days of follow-up, the MACE group continued to exhibit larger left atrial diameter, left ventricular end-systolic dimension (LVESD), left ventricular end-diastolic volume (LVEDV), and left ventricular end-systolic volume (LVESV), as well as reduced LVEF (all *p* < 0.05) (Supplementary Table 3).

The predictive value of serial HBP measurements for LVEF at 180 ± 15 days is presented in Supplementary Table 4. Later HBP measurements (HBP_48h_: *β* = −0.257, *p* = 0.015; HBP_72h_: *β* = −0.364, *p* < 0.001) demonstrated significant inverse associations with LVEF, whereas the HBP_0h_ and HBP_24h_ levels showed no predictive value (all *p* > 0.05). These findings were corroborated by multivariate analysis (Supplementary Table 5), in which HBP_72h_ (*β* = −0.291, *p* = 0.005) remained independently associated with reduced LVEF after adjustment for clinical confounders.

### Association between plasma HBP levels and traditional myocardial injury markers

The correlation matrix of serial measurements of HBP, CK, CK-MB, and hs-cTnI at different time points post-admission is presented in Table 2. At admission, HBP levels showed no significant correlation with myocardial injury markers. A significant positive correlation between HBP and hs-cTnI emerged at 24 h (*r* = 0.41, *p* = 0.040) and further strengthened by 72 h (*r* = 0.56, *p* = 0.011). No significant correlations were observed between HBP and CK or CK-MB at any time point.

**Table 2.** Spearman correlations between HBP and other biomarkers at matched time points.

Biomarkers	Admission		24h		48h		72h	
	r	*P*	r	*P*	r	*P*	r	*P*
hs-CRP	0.07	0.839	0.20	0.160	0.20	0.060	0.55	<0.001
CK	0.03	0.676	−0.05	0.305	−0.02	0.538	0.10	0.459
CK-MB	−0.01	0.956	−0.01	0.858	−0.01	0.684	0.24	0.271
hs-cTnI	0.01	0.886	0.41	0.040	0.50	0.039	0.56	0.011

Correlations were calculated between HBP and the respective biomarkers measured at the same time point. Data are presented as Spearman’s correlation coefficients (r) and *P* values.

HBP: heparin binding protein; hs-CRP: high sensitivity C reactive protein; CK: creatine kinase; CK-MB: creatine kinase-MB isoenzyme; hs-cTnI: high sensitivity cardiac troponin I.

### Association between plasma HBP levels and inflammatory markers

Temporal correlations between HBP and hs-CRP levels are also summarized in Table 2. HBP levels at admission showed minimal correlation with hs-CRP (*r* = 0.07, *p* = 0.839). Although correlation coefficients increased numerically at 24 h and 48 h, these associations did not reach statistical significance (*p* > 0.05). Notably, a strong and statistically significant correlation was observed at 72 h (*r* = 0.55, *p* < 0.001).

### Association between baseline HBP levels and the risk of MACE during long-term follow-up

Forty-six patients (21.4%) experienced MACE during a mean follow-up of 1.5 years (IQR 0.9, 1.9). Among the individual MACE components, hospitalization for HF was the most frequent event (*n* = 27, 12.6%), followed by hospitalization for unstable angina (*n* = 8, 3.7%), all-cause mortality (*n* = 6, 2.8%), recurrent MI (*n* = 3, 1.4%), and stroke (*n* = 2, 0.9%). Patients who developed MACE had higher plasma levels of HBP at 48 h and 72 h compared to those without MACE (*p* < 0.05, Figure 2A). The Cox proportional hazards models revealed varying associations between HBP levels at different time points and MACE risk in patients with STEMI (Table 3). Neither HBP_0h_ nor HBP_24h_ showed significant associations with MACE, whether analyzed as continuous variables (per 1 SD increase) or by quartiles, in both unadjusted and adjusted models (all *p* > 0.05). The strongest association was observed at HBP_72h_. Each 1-SD increase in HBP significantly predicted MACE across all models (fully adjusted HR 1.010; 95% CI 1.002, 1.019; *p* = 0.017). Patients in the highest quartile had a markedly higher risk of MACE compared to those in the lowest quartile (fully adjusted HR 5.533; 95% CI 1.476, 19.242; *p* = 0.011). Additionally, patients with high HBP_72h_ exhibited more severe Killip class, despite the four groups being well matched for cardiovascular risk factors (Supplementary Table 6). However, none of the metrics assessing changes in HBP over time showed significant associations with MACE (Supplementary Table 7).

**Table 3.** Cox models for heparin-binding protein with MACE in patients with STEMI.

	Unadjusted HR (95% CI)	*P* value	Adjusted for model 1 HR (95% CI)	*P* value	Adjusted for model 2 HR (95% CI)	*P* value
HBP at admission						
Continuous per 1 SD increase	1.003 (0.999, 1.008)	0.175	1.003 (0.999, 1.008)	0.186	1.001 (0.996, 1.007)	0.661
Quartile 1	1 (reference)		1 (reference)		1 (reference)	
Quartile 2	1.131 (0.499, 2.564)	0.769	1.244 (0.544, 2.847)	0.605	0.926 (0.381, 2.250)	0.865
Quartile 3	0.633 (0.245, 1.633)	0.344	0.672 (0.260, 1.739)	0.413	1.158 (0.485, 2.768)	0.741
Quartile 4	1.576 (0.731, 3.397)	0.246	1.548 (0.718, 3.338)	0.265	0.620 (0.227, 1.693)	0.351
HBP 24h						
Continuous per 1 SD increase	1.003 (0.998, 1.009)	0.260	1.002 (0.996, 1.008)	0.457	1.005 (0.999, 1.012)	0.128
Quartile 1	1 (reference)		1 (reference)		1 (reference)	
Quartile 2	0.982 (0.409, 2.360)	0.968	0.887 (0.368, 2.135)	0.897	0.879 (0.351, 2.210)	0.783
Quartile 3	1.388 (0.616, 3.129)	0.429	1.278 (0.561, 2.912)	0.559	0.635 (0.242, 1.668)	0.357
Quartile 4	1.133 (0.489, 2.625)	0.771	1.047 (0.451, 2.430)	0.916	0.996 (0.425, 2.331)	0.992
HBP 48h						
Continuous per 1 SD increase	**1.006 (1.001, 1.011)**	**0.017**	**1.006 (1.001, 1.011)**	**0.010**	**1.008 (1.002, 1.014)**	**0.008**
Quartile 1	1 (reference)		1 (reference)		1 (reference)	
Quartile 2	0.862 (0.333, 2.236)	0.761	0.862 (0.332, 2.239)	0.760	0.738 (0.270, 2.019)	0.554
Quartile 3	0.760 (0.283, 2.040)	0.586	0.698 (0.259, 1.885)	0.479	0.673 (0.241, 1.880)	0.449
Quartile 4	**2.861 (1.315, 6.224)**	**0.008**	**3.199 (1.458, 7.020)**	**0.004**	2.326 (0.984, 5.500)	0.055
HBP 72h						
Continuous per 1 SD increase	**1.010 (1.004, 1.016)**	**0.001**	**1.010 (1.005, 1.016)**	**0.001**	**1.010 (1.002, 1.019)**	**0.017**
Quartile 1	1 (reference)		1 (reference)		1 (reference)	
Quartile 2	**4.019 (1.121, 14.417)**	**0.033**	**3.660 (1.016, 13.180)**	**0.047**	3.404 (0.917, 12.637)	0.067
Quartile 3	3.591 (0.988, 13.053)	0.052	3.227 (0.883, 11.787)	0.076	2.926 (0.768, 11.148)	0.116
Quartile 4	**8.536 (2.552, 28.551)**	**0.001**	**8.257 (2.468, 27.632)**	**0.001**	**5.533 (1.476, 19.242)**	**0.011**

Model 1 was adjusted for age and sex; Model 2 was adjusted for model 1 and body mass index, heart rate, systolic blood pressure, smoking, history of hypertension, history of diabetes mellitus, Killip grade, white blood cells, peak of high-sensitivity-C-reactive protein, estimated glomerular filtration rate and peak of high-sensitivity Troponin I. Bold used to highlight those *P* values < 0.05.

STEMI: ST-segment elevation myocardial infarction; HBP: heparin binding protein; MACE: major adverse cardiac events; HR: hazard ratio; CI: confidence interval.

**Figure 3.**
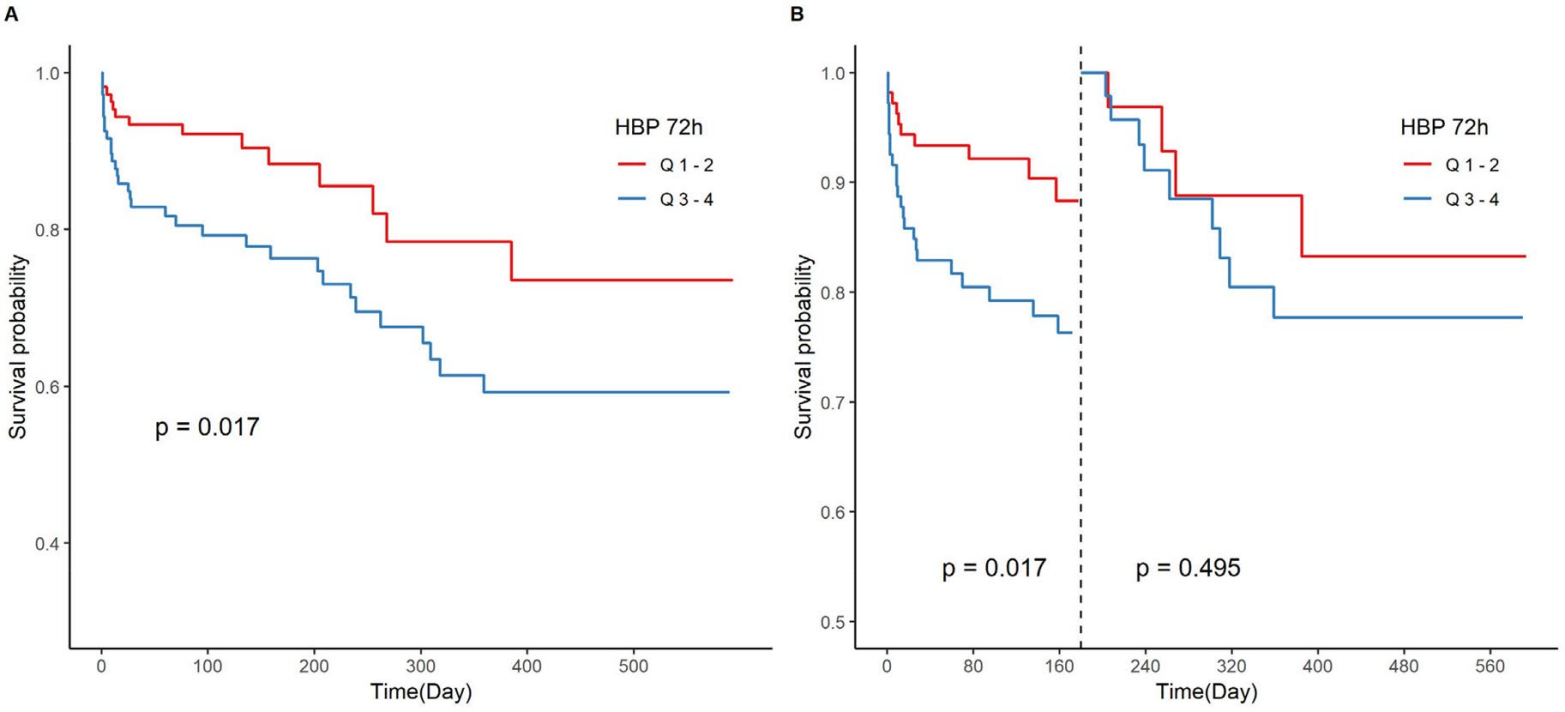
Kaplan–Meier analysis of MACE-free survival according to quartile groups of 72-hour post-pPCI HBP levels. (A) Kaplan–Meier curve of free from follow-up MACE in the HBP quartile1-2 and HBP quartile 3–4 groups. (B) Landmark analysis discriminating between events occurring before and after 180 days of follow-up.

No significant associations were observed between MACE and quartiles of HBP_0h_, HBP_24h_, or HBP_48h_ (all log-rank *p* > 0.05; Supplementary Figure 4) in both overall and landmark analyses. In contrast, Figure 3A shows significantly reduced survival in patients in the highest versus lowest quartiles of HBP_72h_ (log-rank *p* = 0.017). Landmark analysis at 180 days revealed a significant divergence in survival during the first 180 days (*p* = 0.017, Figure 3B), with no statistically significant difference observed beyond this timepoint (post-landmark *p* = 0.495).

### Association between HBP levels and the risk of HF

This study investigated the association between HBP levels and HF risk in patients with STEMI using Fine–Gray competing risk regression analyses. Patients who developed HF exhibited significantly higher plasma HBP_48h_ and HBP_72h_ levels compared to those without HF (*p* < 0.05, Figure 2B). As presented in Supplementary Table 8, at HBP_48h_, patients in the highest quartile exhibited a four-fold increased risk of HF compared to the reference group (HR 3.97; 95% CI 1.14, 13.83; *p* = 0.030). Moreover, the predictive capacity peaked at HBP_72h_, where patients in the highest quartile had an 8.63-fold higher risk of HF (95% CI 1.55, 19.31; *p* < 0.001). These associations remained robust after comprehensive adjustment for demographic characteristics, clinical parameters, and laboratory markers. Although absolute HBP levels demonstrated strong time-dependent predictive value, Supplementary Table 9 indicated that changes in HBP levels over time were not significantly associated with HF (all *p* > 0.05).

### Discrimination and reclassification of MACE

**Figure 4.**
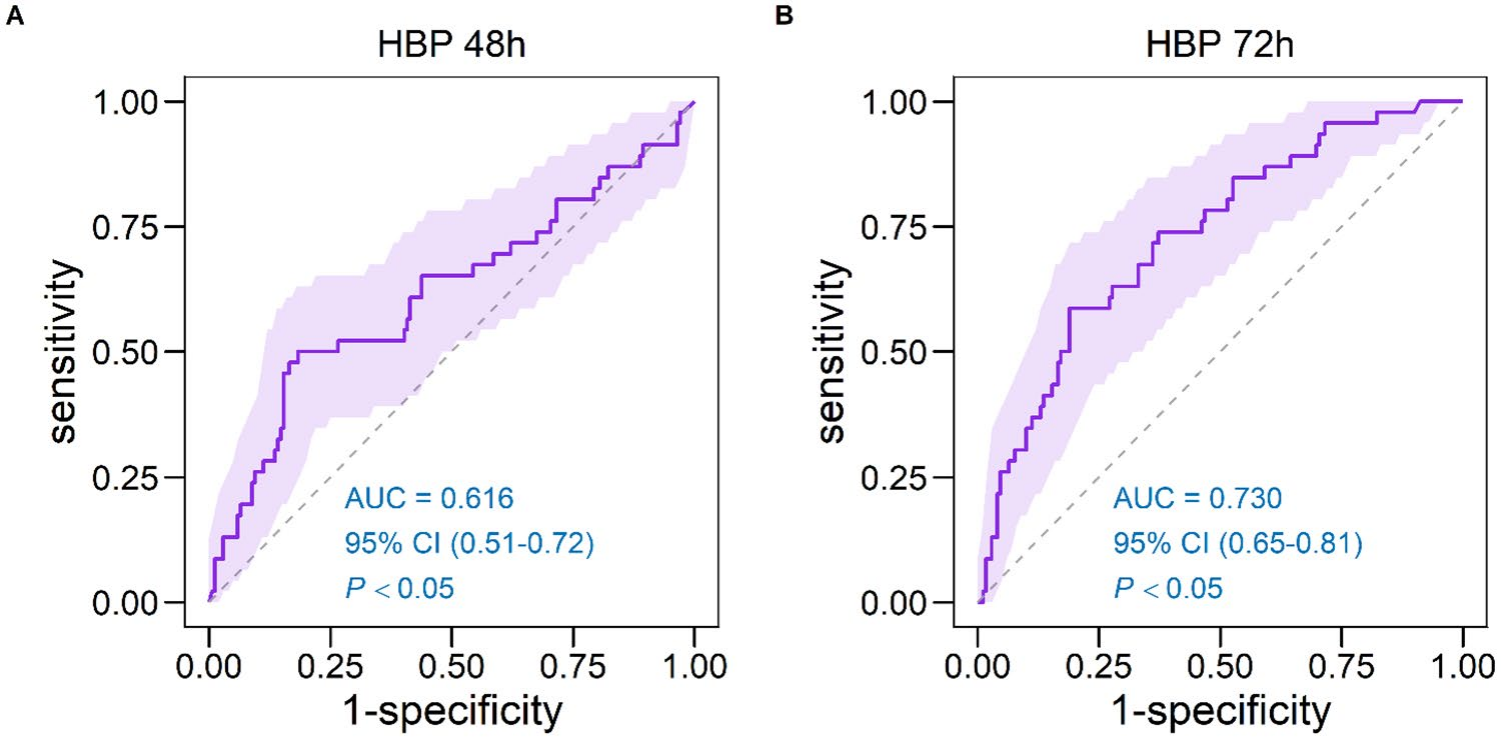
Receiver operating characteristic curve for HBP in major adverse cardiac events. (A) ROC curve analysis of HBP at 48 h; (B) ROC curve analysis of HBP at 72 h. HBP: heparin-binding protein; ROC: receiver operator characteristic curve; AUC: area under the curve.

Supplementary Figure 5 shows that the AUC values for HBP_0h_ and HBP_24h_ were close to 0.5, indicating poor predictive accuracy. Figure 4 and Supplementary Table 10 demonstrate that an HBP_72h_ cut-off level of 29.12 ng/mL predicted MACE after STEMI with 61.9% sensitivity, 78.0% specificity, and an AUC of 0.730.

The reclassification analysis in Table 4 shows that adding HBP_72h_ to a prediction model including peak hs-cTnI significantly improved the prediction of MACE (NRI 0.578; 95% CI 0.263, 0.894; *p* < 0.001). Similar improvements were observed when HBP_72h_ was added to models including hs-CRP (NRI: 0.448; *p* = 0.006), N-terminal pro-brain natriuretic peptide (NT-proBNP) (NRI 0.483; 95% CI 0.164, 0.803; *p* = 0.003), and LVEF (NRI 0.503; 95% CI 0.184, 0.822; *p* = 0.002).

**Table 4.** Improvement of accuracy of risk prediction using combination of conventional parameters and HBP.

	C-Statistic	NRI (95%CI)	*P* value
Peak hs-cTnI	0.796	Ref	NA
Peak hs-cTnI + HBP_72h_	0.853	0.578 (0.263, 0.894)	<0.001
Peak hs-CRP	0.638	Ref	NA
Peak hs-CRP + HBP_72h_	0.715	0.448 (0.128, 0.768)	0.006
Peak NT-proBNP	0.744	Ref	NA
Peak NT-proBNP + HBP_72h_	0.749	0.483 (0.164, 0.803)	0.003
LVEF	0.711	Ref	NA
LVEF + HBP_72h_	0.804	0.503 (0.184, 0.822)	0.002
Peak hs-cTnI + Peak hs-CRP + Peak NT-proBNP + LVEF	0.832	Ref	NA
Peak hs-cTnI + Peak hs-CRP + Peak NT-proBNP + LVEF + HBP_72h_	0.873	0.626 (0.312, 0.940)	<0.001

*P* values NRI are for the difference between old models and new models.

NA: not applicable; Ref: reference; NRI: net reclassification index; hs-cTnI: high sensitivity cardiac troponin I; HBP: heparin binding protein; hs-CRP: high sensitivity C reactive protein; NT-proBNP: N-terminal pro-brain natriuretic peptide; LVEF: left ventricular ejection fraction.

## Discussion

This study demonstrates the distinct kinetics of plasma HBP levels in patients with STEMI, characterized by a pre-pPCI peak followed by a gradual decline over 72 h post-pPCI. This temporal pattern differs from that of conventional inflammatory markers. Most importantly, elevated HBP levels at 72 h independently predicted an increased risk of long-term MACE. These findings position HBP as a potential complementary prognostic biomarker for early risk stratification in patients with STEMI undergoing pPCI.

HBP was selected as a potential biomarker due to its biological role as a protein released by activated neutrophils upon endothelial adhesion or stimulation by circulating bacterial metabolites [17,18]. In healthy individuals, circulating HBP levels are exceedingly low. HBP can disrupt the endothelial cytoskeleton, which compromises the vascular endothelial barrier. This enables WBCs to migrate from capillaries into infected tissues and increases vascular permeability [19]. Studies have shown that HBP also plays a key role in regulating the inflammatory response. By activating monocytes and macrophages, it triggers the release of inflammatory mediators, such as tumor necrosis factor and interferon, thereby amplifying systemic inflammation. This process is closely linked to the development of hypotension and circulatory failure [4]. Abnormal HBP plasma levels have been previously observed in patients with acute kidney injury [20], sepsis [21], pancreatic diseases [22], lung injuries [23], immune system diseases [24], spontaneous bacterial peritonitis [25], and other diseases. Moreover, some studies have suggested that HBP serves as a marker of a worse prognosis in sepsis and acute respiratory distress syndrome [26–28].

However, few studies have explored the relationship between HBP and cardiovascular diseases. Cai et al. [29] employed bioinformatics approaches to characterize HBP networks and revealed significantly higher clustering coefficients in atherosclerosis, myocarditis, myocardial infarction, and myocardial ischemia compared to normal tissues, suggesting HBP’s potential as an inflammatory biomarker in cardiovascular pathology. Ristagno et al. [30] demonstrated that elevated HBP levels predicted early mortality in post-cardiac arrest patients (AUC 0.74). Pan et al. [31] reported that elevated coronary sinus HBP levels were useful in predicting myocardial injury-related cardiogenic shock following cardiac surgery. Additionally, Ipek et al. [32] identified HBP as a potential diagnostic biomarker for AMI, with significantly elevated plasma levels in patients with STEMI compared to healthy controls (18.07 ± 13.99 vs. 10.09 ± 5.29 ng/mL, *p* = 0.018), exhibiting a strong positive correlation with TIMI risk scores (*r* = 0.651, *p* < 0.001). Our findings are consistent with these prior studies but extend the existing evidence on HBP in cardiovascular contexts. To our knowledge, this is the first study to systematically characterize HBP temporal dynamics and establish its independent prognostic value in patients with STEMI undergoing pPCI.

Following AMI, ventricular remodeling is characterized by changes at the molecular, structural, geometrical, and functional levels that determine progression to HF and adverse outcomes. Inflammation plays a key role in wound healing and scar formation, affecting ventricular remodeling. In previous studies, CRP [33], WBC [34], neutrophils, neutrophil to lymphocyte ratio, MG53 [35], BAFF [36], and IL-34 [37] were reported as prognostic markers of AMI. Our findings demonstrate that plasma HBP levels peak early post-pPCI (mean, 43.43 ng/mL at admission) before gradually declining over 72 h (19.54 ng/mL), a pattern contrasting sharply with the delayed response of hs-CRP. Numerous experimental studies have underscored the pathophysiological significance of activated neutrophils in cardiac ischemia-reperfusion injury, where neutrophil accumulation and the release of cytotoxic products exacerbate cardiomyocyte death and contribute to HF development [38,39]. It is plausible that accumulating HBP contributes to coronary endothelial cell injury and myocardial edema formation [40,41]. Notably, coronary HPB release has been observed alongside the coronary release of heart-type fatty acid-binding protein, an early and sensitive biomarker of myocardial injury [42].

The selected sampling time points (0, 24, 48, and 72 h) were explicitly chosen to capture the dynamic evolution of the inflammatory cascade. This schedule encompasses the baseline phase, the peak inflammatory response typically occurring within the first 24–48 h post-pPCI, and the subsequent trajectory towards resolution or persistence of inflammation by 72 h. The persistence of elevated inflammatory markers at this later time point is often crucial for prognostic evaluation. Additionally, this temporal window aligns with the kinetic profiles of major myocardial injury biomarkers, thereby facilitating a comprehensive assessment of post-infarction injury and repair. The cumulative incidence of MACE in our cohort (21.4%) over a median follow-up of 1.5 years is consistent with reports involving high-risk STEMI populations, which have documented comparable composite event rates of approximately 21.0% [43]. With respect to individual components, the observed incidence of new-onset HF rate was 12.6%. This finding closely aligns with data from large-scale cohorts such as the SWEDEHEART registry, which reported HF rates of approximately 12% within the first year post-MI [44], while other studies have reported rates ranging from 10% to 20% [45–47]. In the present study, we therefore specifically explored the association between HBP and HF in greater detail. This focused analysis was justified by the fact that HF hospitalization accounted for the majority (58.7%) of composite adverse events, providing adequate statistical power for robust analysis. By contrast, the relatively low incidence of other individual endpoints precluded separate multivariable analyses. Moreover, given the biological role of HBP in promoting vascular leakage, it represents a particularly relevant biomarker in the pathophysiology of HF.

Additionally, the predictive value of HBP appears stronger for 180-day outcomes than for long-term prognosis in patients with STEMI. This may reflect its pathophysiological specificity. As a neutrophil-derived alarmin, HBP peaks during the critical window of ischemia-reperfusion injury, closely mirroring acute-phase processes, such as microvascular dysfunction and inflammatory storm, which determine early outcomes. Its short half-life makes it an ideal marker for acute endothelial injury and neutrophil activation [48], which drive 180-day events such as early ventricular remodeling and acute HF. However, long-term prognosis depends more on chronic fibrosis and adaptive mechanisms that develop beyond the acute activity period of HBP, which explains its attenuated predictive value over time. This temporal dichotomy highlights the unique role of HBP in acute risk stratification while underscoring the need for complementary biomarkers to assess chronic remodeling. In addition, in acute settings, the selection of patients is performed based on their symptoms and electrocardiogram findings. Given that the symptom onset is usually recent in most STEMI cases, early biomarkers such as troponin, CK-MB, and HBP may not reflect the actual burden of the inflammatory reaction.

Notably, the superior predictive value of absolute HBP concentrations over dynamic changes implies that cumulative neutrophil activation burden, rather than its fluctuation, drives adverse outcomes. The HBP_72h_ level emerged as the most robust predictor, with the highest quartile conferring an 8.63-fold increased HF risk. Although the present study demonstrated that increased plasma HBP levels can be predictive of MACE in patients with STEMI, the relatively lower sensitivity and higher specificity suggest that HBP measurement alone may be insufficient for detecting MACE after AMI. However, it may serve as a complementary diagnostic marker. The significant net reclassification improvement observed when HBP was added to conventional biomarkers (hs-cTnI, NT-proBNP, LVEF) supports its incremental prognostic value within contemporary risk prediction models.

This study has some limitations. Initially, as observational research, even after accounting for established prognostic variables in STEMI, there is still a chance of residual confounding. It is also worth noting that heparin administered prior to pPCI could theoretically influence HBP release, as it displaces endothelium-bound HBP into the circulation *via* competitive binding [49]. However, in our study, all patients received a standardized, weight-adjusted heparin dose before PCI in accordance with guideline-recommended protocols. Given the uniform dosing strategy across the cohort, we consider it unlikely that differences in heparin exposure materially biased the observed HBP kinetics. Second, meaningful subgroup analyses are restricted by the moderate sample size, especially for uncommon endpoints like stent thrombosis. Third, to verify the predictive value of HBP in AMI, our data needs external validation in bigger, multiethnic cohorts with a variety of cardiovascular risk profiles. Fourth, causal conclusions about the relationship between myocardial damage and HBP elevation are not made possible by the lack of mechanistic data. To elucidate the pathophysiological function of HBP in AMI, future studies should combine experimental research with clinical cohorts. Given the prognostic potential identified in this study, future research should prioritize multicenter validation and mechanism studies targeting HBP-related pathways, and should investigate whether modulation of HBP-mediated pathways can reduce vascular leakage and improve clinical outcomes in high-risk patients with STEMI.

## Conclusion

In conclusion, this study establishes HBP as robust and clinically useful biomarker that complements existing tools for risk stratification in patients with STEMI. Notably, the distinct dose–response relationship observed across increasing quartiles strengthens the prognostic relevance of HBP. The 72-h time point appears optimal for measurement and may help inform personalized therapeutic decision making. Collectively, these findings underscore the importance of neutrophil-mediated inflammation in determining STEMI outcomes and highlight the potential of HBP to identify high-risk patients who may benefit from targeted anti-inflammatory strategies.

## Definition of study endpoints

### Myocardial infarction

Myocardial infarction is defined as spontaneous ST-segment elevation myocardial infarction or non-ST-segment elevation myocardial infarction according to the fourth universal definition.

Anyone of the following criteria meets the diagnosis of myocardial infarction:

- Detection of a rise and/or fall of cardiac biomarker values (preferably cardiac troponin) with at least one value above the 99th percentile URL and with at least one of the following:
- Symptoms of ischemia
- (Presumed) new significant ST-T wave changes or new LBBB
- Development of pathological Q waves
- Imaging evidence of new loss of viable myocardium or new regional wall motion abnormality
- Identification of an intracoronary thrombus by angiography or autopsy
- Cardiac death with symptoms suggestive of myocardial infarction and presumed new ischemic ECG changes or new LBBB, but death occurred before cardiac biomarkers were obtained, or before cardiac biomarker values would be increased.

## Hospitalization for heart failure

Hospitalization for heart failure (HF) is defined as an event where the patient is admitted to the hospital with a primary diagnosis of HF where the length of stay is at least 24 h, where the patient exhibits new or worsening symptoms of HF (dyspnea, decreased exercise tolerance, fatigue, worsened end-organ perfusion, or volume overload) on presentation, has objective evidence of new or worsening HF, and receives initiation or intensification of treatment specifically for HF.

## Hospitalization for unstable angina

Hospitalization for unstable angina is defined as unscheduled hospitalization for the management of unstable angina, occurring within 24 h of the most recent symptoms. Hospitalization is defined as an admission to an inpatient unit or a visit to an emergency department that results in at least a 24-h stay. This classification requires that 4 separate criteria be met: a) worsening ischemic discomfort; b) unscheduled hospitalization; c) objective evidence of myocardial ischemia; d) negative cardiac biomarkers.

## Stroke

Stroke is defined as an acute symptomatic episode of neurological dysfunction, more than 24 h in duration in the absence of therapeutic intervention or death, due to cerebral, spinal or retinal tissue injury as evidenced by neuroimaging or lumbar puncture. It includes the following subclassifications:

- Ischemic stroke
- Intracerebral hemorrhage
- Stroke of undetermined etiology

## Supplementary Material

2_Clean 3_Supplement.docx

2_Clean 2_Tables.docx

2_Clean 2_Figure.docx

## Data Availability

The datasets generated and/or analyzed during this study are available from the corresponding author upon reasonable request.
