# Supplementary material for: Kinetics and prognostic value of heparin binding protein at the ST-segment-elevation myocardial infarction
Source: Ann Med. 2026 Jan 30;58(1):2622182. doi: 10.1080/07853890.2026.2622182 (PMC12862846; doi:10.1080/07853890.2026.2622182)
Supplement: 2_Clean 3_Supplement.docx [file IANN_A_2622182_SM7505.docx]

***SUPPLEMENTAL MATERIAL***

**ONLINE TABLES**

**Table S1. Definition of study variables**

|  | **Definition** | **Continuous/Categorical/ Binary** |
| --- | --- | --- |
| **Demographics** |  |  |
| Age | Years after birth | Continuous |
| Sex | Female/Male | Binary, Female/Male |
| Body mass index | Body mass index calculated during hospitalization, kg/m^2^ | Continuous |
| Current smoking | Cigarette smoking anytime during the year prior to arrival at your facility | Binary, Yes/No |
| **Medical history** |  |  |
| Hypertension | 1. Previously diagnosed with hypertension; and/or 2. currently on pharmacological therapy for the treatment of hypertension; and/or 3. SBP ≥ 140 mmHg, or DBP ≥ 90 mmHg during hospitalization. | Binary, Yes/No |
| Diabetes | 1. History of diabetes mellitus, regardless of duration of disease or need for antidiabetic agents; and/or 2. the use of any anti-diabetic medications; and/or 3. fasting blood glucose ≥ 7.0 mmol/L, or HbA1c >6.5% during hospitalization. | Binary, Yes/No |
| Chronic kidney disease | 1. Self-reported history of renal failure; 2. eGFR of <60 mL/min/1.73m^2^ as calculated from the 2021 Chronic Kidney Disease Epidemiology Collaboration (CKD-EPI) formula.^1^ | Binary, Yes/No |
| **Physical Examination** |  |  |
| Systolic blood pressure (SBP) | SBP measured on admission | Continuous, in mmHg |
| Diastolic blood pressure (DBP) | DBP measured on admission | Continuous, in mmHg |
| Heart rate | Heart rate measured on admission | Continuous, per 1 standard deviation of beats per minute |
| Killip class | Evaluated on admission  Class I: no heart failure.  Class II: rales/S3 gallop.  Class III: frank pulmonary edema. Class IV: cardiogenic shock. | Categorical, Class I to IV |
| **Laboratory Examination** |  |  |
| White Blood Cells (WBC) | WBC level measured on admission | Continuous, in ×10^9^/L |
| Hemoglobin | Hemoglobin level measured on admission | Continuous, g/L |
| Platelet | blood platelet count measured on admission | Continuous, ×10^9^/L |
| Fasting Plasma Glucose (FPG) | FPG measured on admission | Continuous, in mmol/L |
| Glycosylated hemoglobin (HbA1c) | Admission HbA1c measured within 24 hours of hospitalization | Continuous, in % |
| [Creatinine](javascript:;) | [Creatinine](javascript:;) measured on admission | Continuous, in µmol/L |
| Estimated glomerular filtration rate (eGFR) | Calculated using CKD-EPI Equation | Continuous, per 10 mL/min/1.73m^2^ |
| Triglycerides (TG) | Admission TG measured within 24 hours of hospitalization | Continuous, in mmol/L |
| Total cholesterol (TC) | Admission TC measured within 24 hours of hospitalization | Continuous, in mmol/L |
| High density lipoprotein cholesterol (HDL-C) | Admission HDL-C measured within 24 hours of hospitalization | Continuous, in mmol/L |
| Low density lipoprotein cholesterol (LDL-C) | Admission LDL-C measured within 24 hours of hospitalization | Continuous, in mmol/L |
| Lp(a) | Admission Lp(a) measured within 24 hours of hospitalization | Continuous, in mmol/L |
| High-sensitive cardiac troponin I (hs-cTnI) | Measured at admission and serial time points (24h, 48h, and 72h after pPCI). | Continuous, in pg/mL |
| Creatine kinase (CK) | Measured at admission and serial time points (24h, 48h, and 72h after pPCI). | Continuous, in IU/L |
| Creatine kinase-MB isoenzyme (CK-MB) | Measured at admission and serial time points (24h, 48h, and 72h after pPCI). | Continuous, in ng/mL |
| High sensitivity C reactive protein (hs-CRP) | Measured at admission and serial time points (24h, 48h, and 72h after pPCI). | Continuous, in mg/L |
| Peak hs-cTnI | The highest concentration recorded of hs-cTnI level during the hospital stay | Continuous, in pg/mL |
| Peak CK | The highest concentration recorded of CK level during the hospital stay | Continuous, in IU/L |
| Peak CK-MB | The highest concentration recorded of CK-MB level during the hospital stay | Continuous, in ng/mL |
| Peak hs-CRP | The highest concentration recorded of hs-CRP level during the hospital stay | Continuous, in mg/L |
| Peak N-terminal pro-brain natriuretic peptide (NT-proBNP) | The highest concentration recorded of NT-proBNP levels during the hospital stay | Continuous, in pg/ml |
| **Echocardiography** |  |  |
| Left atrium diameter (LA) | LA were measured on the parasternal long-axis view by M-mode echocardiography. | Continuous, in mm |
| Left ventricular end-diastolic dimension (LVEDD) | LVEDD, and LVESD measured on the parasternal long-axis view by M-mode echocardiography. |  |
| Left ventricular end-systolic dimension (LVESD) |  |  |
| Left ventricular end-diastolic volume (LVEDV) | LVESV, LVEDV, and LVEF were determined by the biplane method of disk summation (modified Simpson rule) | Continuous, in mL |
| Left ventricular end-systolic volume (LVESV) |  |  |
| Left ventricular ejection fraction (LVEF) |  | Continuous, in % |
| **Medication** |  |  |
| Statins | Use of any kind of statins [post-discharge](javascript:;) | Binary, Yes/No |
| Angiotensin converting enzyme inhibitors/angiotensin receptor blockers (ACEIs/ARBs) | Use of any kind of ACEIs/ARBs [post-discharge](javascript:;) | Binary, Yes/No |
| β-blockers | Use of any kind of β-blockers [post-discharge](javascript:;) | Binary, Yes/No |

**Table S2.** Missing rates of study variables and management of the missingness*

| **Variables** | **Missing, No. (%) (n=215)** | **Management of missing data** |
| --- | --- | --- |
| Chronic kidney disease | 2 (0.9) | Imputed using multiple imputation method implemented by R software |
| Hemoglobin | 2 (0.9) |  |
| Fasting glucose | 1 (0.5) |  |
| HbA1c | 3 (1.4) |  |
| CK at admission | 1 (0.5) |  |
| CK at 24h after pPCI | 4 (1.9) |  |
| CK at 48h after pPCI | 6 (2.8) |  |
| CK at 72h after pPCI | 10 (4.7) |  |
| CK-MB at admission | 1 (0.5) |  |
| CK-MB at 24h after pPCI | 4 (1.9) |  |
| CK-MB at 48h after pPCI | 4 (1.9) |  |
| CK-MB at 72h after pPCI | 7 (3.3) |  |
| hs-cTnI at admission | 1 (0.5) |  |
| hs-cTnI at 24h after pPCI | 4 (1.9) |  |
| hs-cTnI at 48h after pPCI | 5 (2.5) |  |
| hs-cTnI at 72h after pPCI | 7 (3.3) |  |
| hs-CRP at admission | 10 (4.7) |  |
| hs-CRP at 24h after pPCI | 18 (8.4) |  |
| hs-CRP at 48h after pPCI | 24 (11.2) |  |
| hs-CRP at 72h after pPCI | 26 (12.1) |  |
| Killip Class | 2 (0.9) | Patients with unclear status for Killip class were imputed based on the status of heart failure or cardiogenic shock at admission. Patients with acute heart failure were identified as Killip class 2-3 and patients with cardiogenic shock were identified as Killip class IV. |

*There were no missing values for the following parameters: age, sex, current smoking, body mass index, systolic blood pressure, diastolic blood pressure, heart rate, history of diabetes mellitus and hypertension, culprit lesion, white blood cell count, platelet count, creatinine (for estimated glomerular filtration rate calculation), triglycerides, total cholesterol, high density lipoprotein cholesterol, low density lipoprotein cholesterol, Lp (a), peak of high-sensitive cardiac troponin I, peak of creatine kinase, peak of creatine kinase-MB, peak of high sensitivity C reactive protein, peak of N-terminal pro-brain natriuretic peptide.

**Table S3. Echocardiography parameters at 180±15 days after percutaneous coronary intervention.**

| Echocardiography | Total (n = 215) | Non-MACE (n = 169) | MACE (n = 46) | *P* value |
| --- | --- | --- | --- | --- |
| LA, mm | 39.69±3.62 | 39.35±3.51 | 40.93±3.76 | 0.01 |
| LVEDD, mm | 50.57±5.24 | 50.18±4.96 | 52.00±6.01 | 0.04 |
| LVESD, mm | 33.93±5.89 | 33.31±5.67 | 36.20±6.18 | <0.01 |
| LVEDV, mL | 122.44±31.16 | 120.17±29.44 | 130.77±35.94 | 0.04 |
| LVESV, mL | 46 (36, 60) | 44 (35, 59) | 52 (44, 66) | <0.01 |
| LVEF, % | 61.22±6.93 | 62.07±6.36 | 58.08±8.04 | <0.01 |

Data were expressed as mean ± standard deviation or median (first quartile, third quartile). Abbreviations: MACE, major adverse cardiac events; PCI, percutaneous coronary intervention; LA, left atrial diameter; LVEDD, left ventricular end-diastolic diameter; LVESD, left ventricular end-systolic diameter; LVEDV, left ventricular end-diastolic volume; LVESV, left ventricular end-systolic volume; LVEF, left ventricular ejection fraction.

**Table S4. Predictability of heparin-binding protein on LVEF at 6 months after PCI**

|  | LVEF at 180±15 days after PCI | | |
| --- | --- | --- | --- |
|  | *β* | *95% CI for β* | *P* value |
| HBP at admission | 0.069 | -0.022, 0.036 | 0.630 |
| HBP 24h | -0.121 | -0.072, 0.019 | 0.247 |
| HBP 48h | **-0.257** | **-0.096, -0.010** | **0.015** |
| HBP 72h | **-0.364** | **-0.099, -0.031** | **<0.001** |

Data are presented as coefficient of regression equation (*β*) with 95% confidence intervals (CI). Δ_1_ HBP was calculated as the level assessed at admission minus at 24 hours after PCI level of HBP; Δ_2_ HBP was calculated as the level assessed at admission minus at 48 hours after PCI level of HBP; Δ_3_ HBP was calculated as the level assessed at admission minus at 72 hours after PCI level of HBP; Abbreviations: HBP, heparin-binding protein; LVEF, left ventricular ejection fraction; PCI, percutaneous coronary intervention; CI, confidence interval.

**Table S5. Predictability of heparin-binding protein on LVEF at 6 months after PCI**

|  | LVEF at 180±15 days after PCI | | |
| --- | --- | --- | --- |
|  | *β* | *95% CI for β* | *P* value |
| HBP 48h |  |  |  |
| Model 1 | **-0.218** | **(-0.089, -0.001)** | **0.044** |
| Model 2 | -0.162 | (-0.080, 0.012) | 0.146 |
| HBP 72h |  |  |  |
| Model 1 | **-0.327** | **(-0.093, -0.023)** | **0.002** |
| Model 2 | **-0.291** | **(-0.087, -0.016)** | **0.005** |

Data are presented as coefficient of regression equation (*β*) with 95% confidence intervals (CI).

Model 1 was adjusted for age and gender; Model 2 was adjusted for model 1 and body mass index, heart rate, systolic blood pressure, smoking, history of hypertension, history of diabetes mellitus, Killip grade, white blood cells, high-sensitivity-C-reactive protein, estimated glomerular filtration rate and peak of high-sensitivity Troponin I. Abbreviations: LVEF, left ventricular ejection fraction; PCI, percutaneous coronary intervention; HBP, heparin-binding protein.

**Table S6. Clinical characteristics of patients in the quartile based on the value of HBP at 72 hours after PCI**

|  | HBP 72h quartile 1  [n=53] | HBP 72h quartile 2  [n=55] | HBP 72h quartile 3  [n=53] | HBP 72h quartile 4  [n=54] | *P* value |
| --- | --- | --- | --- | --- | --- |
| **Demographic and risk factors** |  |  |  |  |  |
| Age, year | 61.77±13.34 | 64.16±13.08 | 66.81±10.21 | 63.93±11.95 | 0.21 |
| Male, n (%) | 41 (77.36) | 45 (81.82) | 45 (84.91) | 39 (72.22) | 0.40 |
| Current smoking, n (%) | 12 (22.64) | 9 (16.36) | 13 (24.53) | 17 (31.48) | 0.47 |
| BMI, kg/m^2^ | 25.58±4.11 | 24.87±3.52 | 24.11±4.46 | 24.30±5.16 | 0.53 |
| Admission heart rate, bpm | 81 (74, 89) | 80 (70, 95) | 85 (79, 92) | 81 (72, 97) | 0.21 |
| Admission SBP, mmHg | 128 (109, 142) | 124 (109, 149) | 122 (107, 133) | 129 (107, 145) | 0.29 |
| Admission DBP, mmHg) | 79 (68, 88) | 76 (70, 87) | 75 (67, 85) | 76 (66, 87) | 0.87 |
| **Comorbidities** |  |  |  |  |  |
| Diabetes mellitus, n (%) | 13 (24.53) | 14 (25.45) | 13 (24.53) | 20 (37.04) | 0.39 |
| Hypertension, n (%) | 41 (77.36) | 38 (69.09) | 33 (62.26) | 35 (64.81) | 0.36 |
| Chronic kidney disease, n (%) | 3 (5.66) | 2 (3.64) | 1 (1.89) | 9 (16.67) | 0.01 |
| **Killip classification, n (%)** |  |  |  |  | 0.03 |
| I | 48 (90.57) | 48 (87.27) | 46 (86.79) | 44 (81.48) |  |
| II | 5 (9.43) | 3 (5.45) | 3 (5.66) | 2 (3.70) |  |
| III-IV | 0 (0) | 4 (7.28) | 4 (7.55) | 8 (14.81) |  |
| **Culprit lesion, n (%)** |  |  |  |  | 0.37 |
| LAD | 22 (41.51) | 32 (58.18) | 25 (47.17) | 29 (53.70) |  |
| LCX | 12 (22.64) | 11 (20.00) | 10 (18.87) | 10 (18.52) |  |
| RCA | 19 (35.85) | 12 (21.82) | 18 (33.96) | 15 (27.78) |  |
| **Laboratory values** |  |  |  |  |  |
| WBC, ×10^9^/L | 9.86 (8.46, 11.77) | 10.92 (8.93, 12.96) | 10.69 (8.58, 12.96) | 11.20 (9.18, 14.61) | 0.07 |
| Hemoglobin, g/L | 142 (127, 154) | 139 (126, 150) | 137 (125, 146) | 137 (123, 149) | 0.67 |
| Platelet, ×10^9^/L | 216 (187, 240) | 210 (173, 251) | 201 (168, 254) | 232 (187, 263) | 0.09 |
| Fasting glucose, mmol/L | 6.61 (5.87, 7.82) | 6.59 (5.63, 8.48) | 6.01 (5.62, 7.47) | 7.87 (6.02, 11.20) | 0.01 |
| HbA1c, % | 5.90 (5.50, 6.70) | 5.90 (5.60, 6.80) | 5.80 (5.50, 6.60) | 6.20 (5.70, 8.10) | 0.05 |
| Creatine, μmol/L | 74.00 (68.00, 84.00) | 81.00 (68.00, 97.00) | 81.00 (66.00, 96.00) | 80.00 (67.00, 98.00) | 0.47 |
| eGFR, mL/minute/1.73m^2^ | 91.90 (75.20, 100.40) | 80.90 (66.10, 95.60) | 82.00 (69.20, 93.50) | 84.50 (66.90, 97.90) | 0.30 |
| Triglyceride, mmol/L | 1.79 (1.17, 2.41) | 1.57 (1.02, 2.12) | 1.55 (1.13, 2.16) | 1.68 (1.25, 2.28) | 0.49 |
| Total cholesterol, mmol/L | 4.99 (4.36, 5.60) | 4.96 (3.76, 5.74) | 4.85 (4.20, 5.64) | 4.67 (4.02, 5.54) | 0.85 |
| HDL-C, mmol/L | 0.94 (0.85, 1.20) | 1.01 (0.90, 1.21) | 1.10 (0.92, 1.23) | 1.03 (0.92, 1.18) | 0.38 |
| LDL-C, mmol/L | 3.13 (2.77, 3.65) | 3.04 (2.11, 3.64) | 3.06 (2.57, 3.59) | 2.84 (2.49, 3.62) | 0.81 |
| Lp(a), mmol/L | 0.13 (0.07, 0.26) | 0.16 (0.06, 0.37) | 0.25 (0.09, 0.40) | 0.13 (0.07, 0.42) | 0.34 |
| Peak NT-proBNP, pg/ml | 900.70 (429.30, 1929.00) | 1337.0 (499.20, 2505.00) | 1171.0 (507.40, 4625.00) | 1424.0 (597.60, 3776.70) | 0.38 |
| Peak hs-CRP, mg/L | 36.68 (14.12, 139.25) | 75.44 (22.07, 161.56) | 75.19 (32.41, 164.82) | 108.38 (24.13, 171.28) | 0.04 |
| Peak CK, IU/L | 736.00 (433.00, 1738.00) | 726.00 (336.00, 2079.00) | 1152.0 (520.00, 1727.00) | 1385.5 (600.00, 2128.00) | 0.02 |
| Peak CKMB, ng/mL | 45.10 (24.50, 118.90) | 54.60 (12.70, 139.60) | 74.40 (25.90, 131.50) | 71.85 (28.10, 136.60) | 0.04 |
| Peak cTnI, pg/mL | 18454.0 (6935.0, 34910.0) | 24202.0 (7268.6, 53218.0) | 28324.0 (14541.0, 53864.0) | 44681.0 (18026.0, 66936.0) | 0.01 |
| **Medication** |  |  |  |  |  |
| ACEIs/ARBs/ARNIs, n (%) | 40 (75.47) | 40 (72.73) | 41 (77.36) | 40 (74.07) | 0.95 |
| β-blockers, n (%) | 39 (73.58) | 37 (67.27) | 40 (75.47) | 37 (68.52) | 0.75 |
| Statins, n (%) | 50 (94.34) | 51 (92.73) | 50 (94.34) | 49 (90.74) | 0.87 |
| MRAs, n (%) | 5 (9.43) | 7 (12.73) | 3 (5.66) | 9 (16.67) | 0.31 |

Values are mean ± SD, n (%), or median (first quartile, third quartile). The cut-off values for the quartiles of HBP_72h_ were: Q1 (HBP_72h_ < 14.03 ng/mL), Q2 (14.03 ng/mL≤ HBP_72h_ < 23.80 ng/mL), Q3 (23.80 ng/mL≤ HBP_72h_ < 44.51 ng/mL), and Q4 (HBP_72h_ *≥* 44.51 ng/mL). BMI, body mass index; HR, heart rate; SBP, systolic blood pressure; DBP, diastolic blood pressure; LAD, left anterior descending; LCX, left circumflex coronary; RCA, right coronary artery; WBC, white blood cell; HbA1c, glycated hemoglobin; eGFR, estimated glomerular filtration; NT-proBNP, N-terminal pro-brain natriuretic peptide; LDL-C, low-density lipoprotein cholesterol; HDL-C, high-density lipoprotein cholesterol; Lp(a), lipoprotein(a); hs-CRP, high sensitivity C reactive protein; CK, creatine kinase; CK-MB, creatine kinase-MB isoenzyme; cTnl, Cardiac troponin I; ACEIs, angiotensin converting enzyme inhibitors; ARBs, angiotensin receptor blockers; ARNI, angiotensin receptor/neprilysin inhibitor; MRAs, mineralocorticoid receptor antagonists.

**Table S7.** **Cox models for heparin-binding protein with MACE in patients with STEMI**

|  | Unadjusted  HR (95% CI) | *P* value | Adjusted for model 1 HR (95% CI) | *P* value | Adjusted for model 2  HR (95% CI) | *P* value |
| --- | --- | --- | --- | --- | --- | --- |
| **MACE** | | | | | | |
| ∆_1_ HBP | 1.001 (0.997, 1.005) | 0.617 | 1.001 (0.997, 1.006) | 0.516 | 1.000 (0.995, 1.006) | 0.874 |
| ∆_2_ HBP | 0.999 (0.995, 1.004) | 0.788 | 0.999 (0.995, 1.004) | 0.683 | 0.998 (0.993, 1.003) | 0.378 |
| ∆_3_ HBP | 0.999 (0.994, 1.004) | 0.645 | 0.999 (0.993, 1.004) | 0.959 | 1.000 (0.994, 1.006) | 0.938 |
| ∆_4_ HBP | 0.996 (0.990, 1.003) | 0.229 | 0.995 (0.988, 1.001) | 0.100 | 0.994 (0.986, 1.001) | 0.101 |
| ∆_5_ HBP | 0.995 (0.989, 1.002) | 0.168 | 0.995 (0.989, 1.001) | 0.101 | 0.999 (0.991, 1.006) | 0.755 |
| ∆_6_ HBP | 0.999 (0.992, 1.007) | 0.829 | 1.000 (0.992, 1.007) | 0.922 | 1.003 (0.997, 1.009) | 0.286 |

Δ_1_ HBP was calculated as the level assessed at admission minus at 24 hours after pPCI level of HBP; Δ_2_ HBP was calculated as the level assessed at admission minus at 48 hours after pPCI level of HBP; Δ_3_ HBP was calculated as the level assessed at admission minus at 72 hours after pPCI level of HBP; Δ_4_ HBP was calculated as the level assessed at 24 hours after PCI minus at 48 hours after PCI level of HBP; Δ_5_ HBP was calculated as the level assessed at 24 hours after PCI minus at 72 hours after PCI level of HBP; Δ_6_ HBP was calculated as the level assessed at 48 hours after PCI minus at 72 hours after PCI level of HBP. Model 1 was adjusted for age and gender; Model 2 was adjusted for model 1 and body mass index, heart rate, systolic blood pressure, smoking, history of hypertension, history of diabetes mellitus, Killip grade, white blood cells, peak of high-sensitivity-C-reactive protein, estimated glomerular filtration rate and peak of high-sensitivity Troponin I. Abbreviations: HBP, heparin binding protein; MACE, major adverse cardiac events; HF, heart failure; HR, hazard ratio; CI, confidence interval.

**Table S8.** **Association between heparin-binding protein and risk of heart failure using Fine & Gray models for competing risk**

|  | Unadjusted  HR (95% CI) | *P* value | Adjusted for model 1 HR (95% CI) | *P* value | Adjusted for model 2  HR (95% CI) | *P* value |
| --- | --- | --- | --- | --- | --- | --- |
| HBP at admission | | | | | | |
| Continuous Per 1 increase | 1.001 (0.998, 1.011) | 0.602 | 1.003 (0.993, 1.013) | 0.714 | 1.004 (0.992, 1.007) | 0.742 |
| Quartile 1 | 1 (reference) |  | 1 (reference) |  | 1 (reference) |  |
| Quartile 2 | 1.241 (0.419, 3.624) | 0.703 | 1.474 (0.474, 4.627) | 0.505 | 1.356 (0.431, 4.290) | 0.595 |
| Quartile 3 | 1.003 (0.325, 3.083) | >0.99 | 1.062 (0.330, 3.389) | 0.613 | 0.885 (0.255, 2.968) | 0.891 |
| Quartile 4 | 1.386 (0.491, 3.927) | 0.526 | 1.298 (0.468, 3.638) | 0.605 | 0.750 (0.177, 3.063) | 0.751 |
| HBP 24h | | | | | | |
| Continuous Per 1 increase | 1.011 (1.003, 1.014) | 0.005 | 1.006 (1.002, 1.010) | 0.010 | 1.013 (1.004, 1.021) | 0.018 |
| Quartile 1 | 1 (reference) |  | 1 (reference) |  | 1 (reference) |  |
| Quartile 2 | 0.992 (0.249, 3.901) | 0.988 | 0.887 (0.228, 3.435) | 0.873 | 0.809 (0.201, 3.281) | 0.763 |
| Quartile 3 | 2.238 (0.696, 7.179) | 0.169 | 2.173 (0.672, 7.092) | 0.192 | 1.653 (0.472, 5.832) | 0.427 |
| Quartile 4 | 2.453 (0.779, 7.685) | 0.121 | 2.207 (0.711, 6.880) | 0.166 | 2.376 (0.625, 8.981) | 0.202 |
| HBP 48h | | | | | | |
| Continuous Per 1 increase | **1.008 (1.001, 1.012)** | **<0.001** | **1.006 (1.001, 1.011)** | **<0.001** | **1.008 (1.002, 1.016)** | **<0.001** |
| Quartile 1 | 1 (reference) |  | 1 (reference) |  | 1 (reference) |  |
| Quartile 2 | 0.722 (0.163, 3.176) | 0.661 | 0.672 (0.162, 2.819) | 0.581 | 0.478 (0.140, 1.479) | 0.194 |
| Quartile 3 | 0.731 (0.173, 3.190) | 0.671 | 0.593 (0.139, 2.505) | 0.469 | 0.513 (0.101, 2.520) | 0.409 |
| Quartile 4 | **4.731 (1.615, 13.824)** | **0.004** | **4.979 (1.828, 13.520)** | **0.002** | **3.966 (1.144, 13.832)** | **0.030** |
| HBP 72h | | | | | | |
| Continuous Per 1 increase | **1.010 (1.004, 1.016)** | **0.003** | **1.011 (1.005, 1.017)** | **0.003** | 1.010 (1.002, 1.019) | 0.207 |
| Quartile 1 | 1 (reference) |  | 1 (reference) |  | 1 (reference) |  |
| Quartile 2 | **5.328 (1.923, 13.750)** | **<0.001** | **2.850 (1.558, 13.721)** | **<0.001** | **4.643 (2.650, 12.243)** | **<0.001** |
| Quartile 3 | **6.747 (1.621, 14.561)** | **<0.001** | **7.586 (1.185, 15.730)** | **<0.001** | **9.068 (3.541, 17.224)** | **<0.001** |
| Quartile 4 | **8.022 (3.235, 18.402)** | **<0.001** | **8.510 (1.609, 20.632)** | **<0.001** | **8.634 (1.554, 19.311)** | **<0.001** |

Model 1 was adjusted for age and gender; Model 2 was adjusted for model 1 and body mass index, heart rate, systolic blood pressure, smoking, history of hypertension, history of diabetes mellitus, Killip grade, white blood cells, high-sensitivity-C-reactive protein, estimated glomerular filtration rate and peak of high-sensitivity Troponin I. Bold used to highlight those P values < 0.05. Abbreviations: STEMI, ST-segment elevation myocardial infarction; HBP, heparin binding protein; HR, hazard ratio; CI, confidence interval.

**Table S9.** **Association between heparin-binding protein and risk of heart failure using Fine & Gray models for competing risk**

|  | Unadjusted  HR (95% CI) | *P* value | Adjusted for model 1 HR (95% CI) | *P* value | Adjusted for model 2  HR (95% CI) | *P* value |
| --- | --- | --- | --- | --- | --- | --- |
| **HF** | | | | | | |
| ∆_1_ HBP | 1.003 (0.997, 1.008) | 0.303 | 1.003 (0.997, 1.008) | 0.375 | 1.006 (0.999, 1.013) | 0.092 |
| ∆_2_ HBP | 1.005 (0.999, 1.010) | 0.075 | 1.006 (1.000, 1.011) | 0.052 | 1.007 (0.999, 1.015) | 0.061 |
| ∆_3_ HBP | 1.001 (0.996, 1.011) | 0.311 | 1.005 (0.997, 1.013) | 0.225 | 1.005 (0.996, 1.014) | 0.286 |
| ∆_4_ HBP | 0.995 (0.987, 1.004) | 0.275 | 0.994 (0.986, 1.002) | 0.162 | 0.994 (0.985, 1.003) | 0.224 |
| ∆_5_ HBP | 1.001 (0.993, 1.009) | 0.830 | 0.999 (0.992, 1.007) | 0.888 | 1.004 (0.995, 1.012) | 0.414 |
| ∆_6_ HBP | 1.005 (0.998, 1.011) | 0.172 | 1.005 (0.998, 1.012) | 0.188 | 1.006 (0.999, 1.012) | 0.078 |

Δ_1_ HBP was calculated as the level assessed at admission minus at 24 hours after pPCI level of HBP; Δ_2_ HBP was calculated as the level assessed at admission minus at 48 hours after pPCI level of HBP; Δ_3_ HBP was calculated as the level assessed at admission minus at 72 hours after pPCI level of HBP; Δ_4_ HBP was calculated as the level assessed at 24 hours after PCI minus at 48 hours after PCI level of HBP; Δ_5_ HBP was calculated as the level assessed at 24 hours after PCI minus at 72 hours after PCI level of HBP; Δ_6_ HBP was calculated as the level assessed at 48 hours after PCI minus at 72 hours after PCI level of HBP. Model 1 was adjusted for age and gender; Model 2 was adjusted for model 1 and body mass index, heart rate, systolic blood pressure, smoking, history of hypertension, history of diabetes mellitus, Killip grade, white blood cells, peak of high-sensitivity-C-reactive protein, estimated glomerular filtration rate and peak of high-sensitivity Troponin I. Abbreviations: HBP, heparin binding protein; MACE, major adverse cardiac events; HF, heart failure; HR, hazard ratio; CI, confidence interval.

**Table S10.** **ROC analyses sensitivity and specificity at cutoffs for heparin-binding protein**

|  | AUC (95% CI) | | Cut-off | Sensitivity | Specificity | Accuracy |
| --- | --- | --- | --- | --- | --- | --- |
| HBP at admission | 0.568 | 0.400, 0.718 | 37.58 | 0.130 | 0.917 | 0.749 |
| HBP 24h | 0.558 | 0.468, 0.648 | 33.36 | 0.109 | 0.935 | 0.758 |
| HBP 48h | 0.616 | 0.514, 0.719 | 52.32 | 0.500 | 0.853 | 0.767 |
| HBP 72h | 0.730 | 0.649, 0.812 | 29.12 | 0.619 | 0.780 | 0.791 |

Abbreviations: HBP, heparin binding protein; ROC, receiver operator characteristic curve; AUC, area under the curve.

**

Online Figures**

**Figure S1. Flow chat.** Abbreviations: STEMI, ST-segment–elevation myocardial infarction; HBP, heparin binding protein; MACE, major adverse cardiac events.


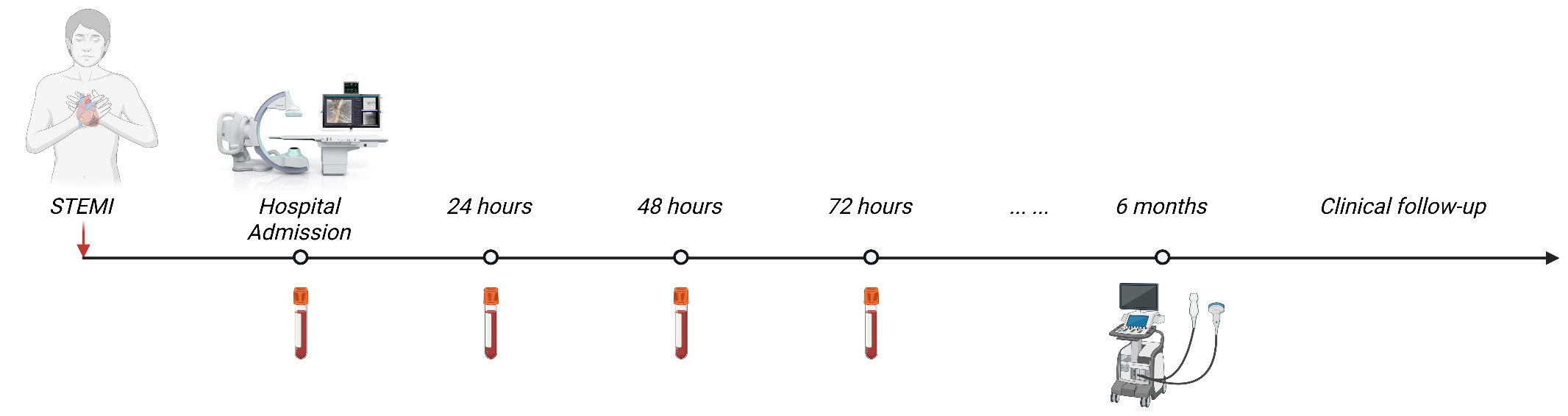


**Figure S2. Study design.** Blood samples were collected at 4 time points: at hospital admission (at the time of coronary angiography), and then at 24 hours, 48 hours, and 72 hours after admission for each patient. Echocardiography was performed on the 1st day and at 180±15 days after percutaneous coronary intervention. Abbreviations: STEMI, ST-segment–elevation myocardial infarction.


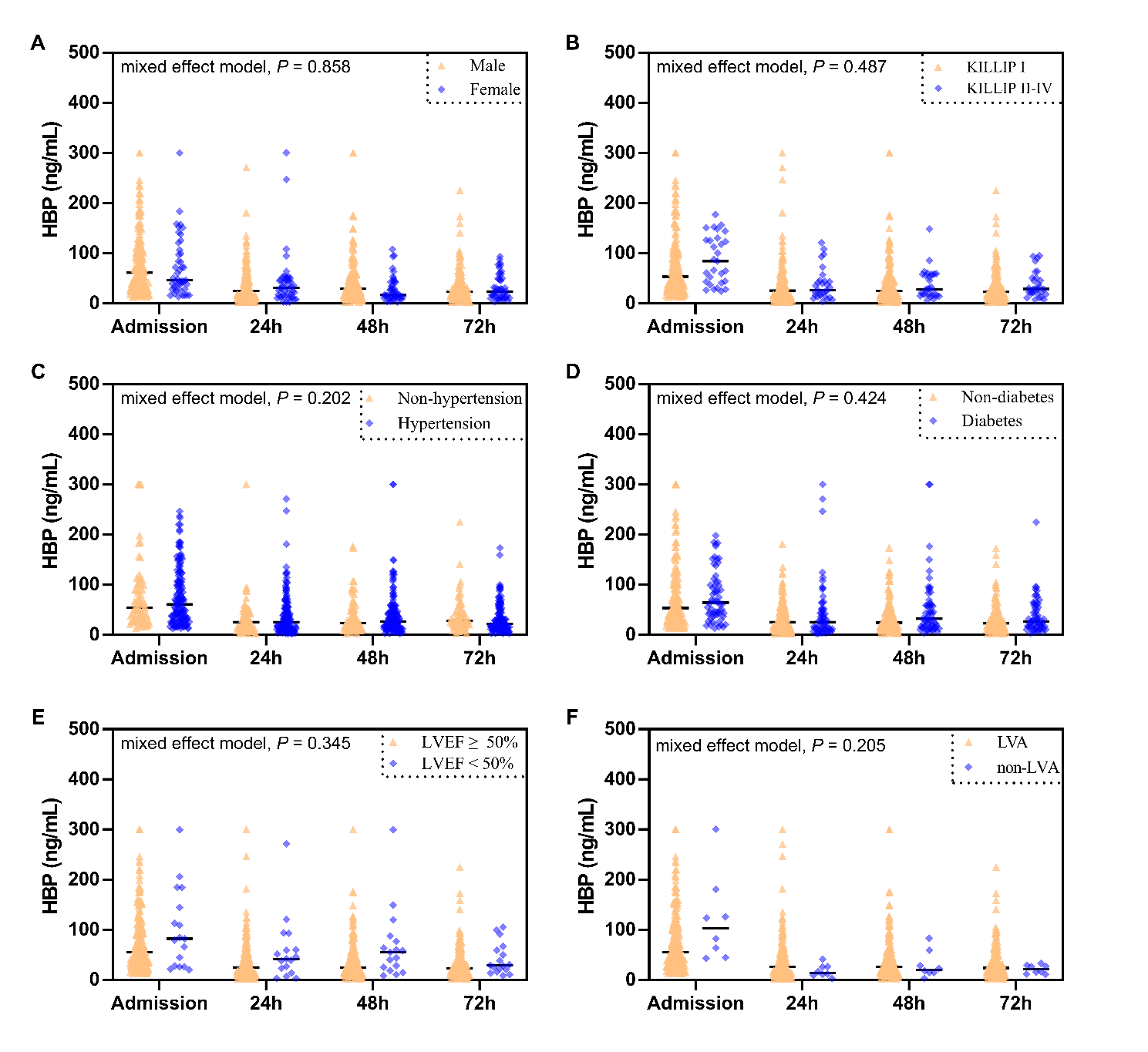
**Figure S3. HBP plasma release kinetics and correlative analysis.** (A) Plasma HBP level according to gender; (B) Plasma HBP level according to Killip grade; (C) Plasma HBP level according to history of hypertension; (D) Plasma HBP level according to history of diabetes mellitus; (E) Plasma HBP level according to left ventricular ejection fraction; (F) Plasma HBP level according to ventricular aneurysm. Abbreviations: HBP, heparin-binding protein; LVEF, left ventricular ejection fraction; LVA, ventricular aneurysm.


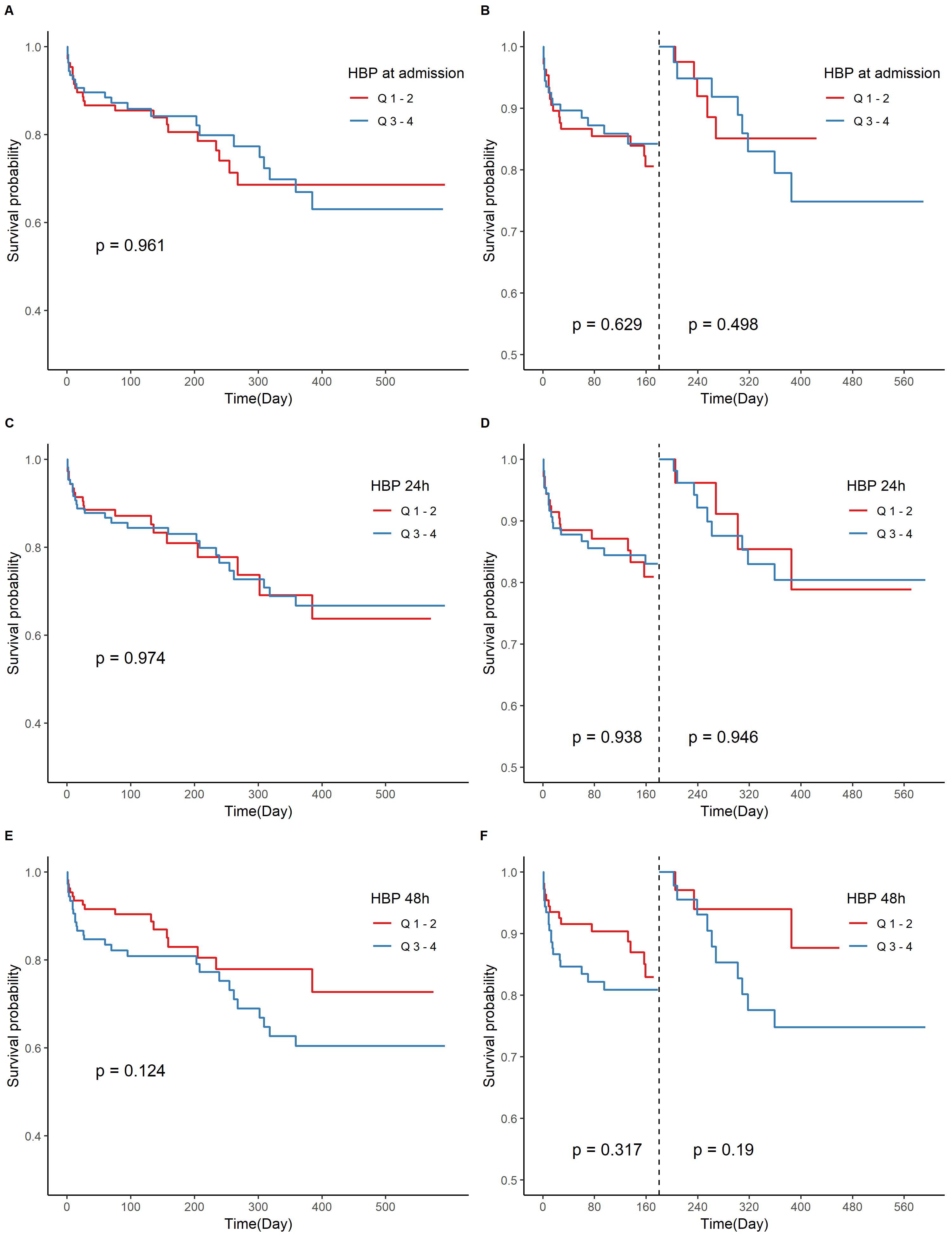
**Figure S4. Kaplan–Meier analysis of MACE-free survival according to quartile groups of HBP levels.** (A) Kaplan–Meier curve of free from follow-up MACE in the HBP quartile1-2 and HBP quartile 3-4 groups at admission; (B) landmark analysis based on admission HBP levels discriminating between events occurring before and after 180 days of follow-up; (C) Kaplan–Meier curve of free from follow-up MACE in the HBP quartile1-2 and HBP quartile 3-4 groups at 48 hours after PCI; (D) landmark analysis based on HBP levels at 48 hours after PCI discriminating between events occurring before and after 180 days of follow-up; (E) Kaplan–Meier curve of free from follow-up MACE in the HBP quartile1-2 and HBP quartile 3-4 groups at 72 hours after PCI; (F) landmark analysis based on HBP levels at 72 hours after PCI discriminating between events occurring before and after 180 days of follow-up. Abbreviations: HBP, heparin-binding protein; PCI, percutaneous coronary intervention.


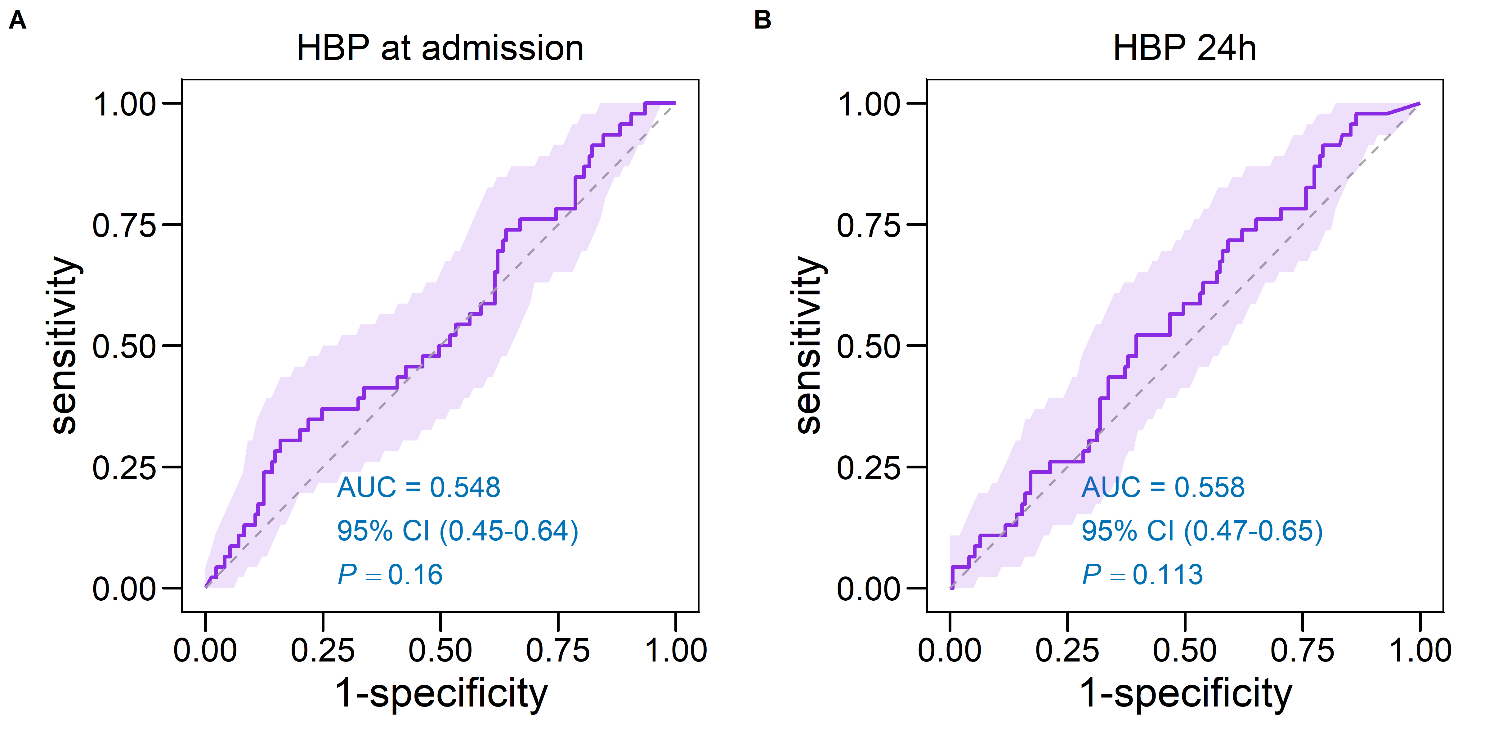
**Figure S5. Receiver operating characteristic curve for HBP in major adverse cardiac events.** (A) ROC curve analysis of HBP at admission; (B) ROC curve analysis of HBP at 24 hours. Abbreviations: HBP, heparin-binding protein; ROC, receiver operator characteristic curve; AUC, area under the curve.

**References:**

1. Levey AS, Stevens LA, Schmid CH, Zhang YL, Castro AF, 3rd, Feldman HI, Kusek JW, Eggers P, Van Lente F, Greene T, et al. A new equation to estimate glomerular filtration rate. *Annals of internal medicine*. 2009;150:604-612. doi: 10.7326/0003-4819-150-9-200905050-00006

2. Lang RM, Bierig M, Devereux RB, Flachskampf FA, Foster E, Pellikka PA, Picard MH, Roman MJ, Seward J, Shanewise JS, et al. Recommendations for chamber quantification: a report from the American Society of Echocardiography's Guidelines and Standards Committee and the Chamber Quantification Writing Group, developed in conjunction with the European Association of Echocardiography, a branch of the European Society of Cardiology. *Journal of the American Society of Echocardiography : official publication of the American Society of Echocardiography*. 2005;18:1440-1463. doi: 10.1016/j.echo.2005.10.005

3. Lang RM, Badano LP, Mor-Avi V, Afilalo J, Armstrong A, Ernande L, Flachskampf FA, Foster E, Goldstein SA, Kuznetsova T, et al. Recommendations for cardiac chamber quantification by echocardiography in adults: an update from the American Society of Echocardiography and the European Association of Cardiovascular Imaging. *Journal of the American Society of Echocardiography : official publication of the American Society of Echocardiography*. 2015;28:1-39.e14. doi: 10.1016/j.echo.2014.10.003

4. Galderisi M, Cosyns B, Edvardsen T, Cardim N, Delgado V, Di Salvo G, Donal E, Sade LE, Ernande L, Garbi M, et al. Standardization of adult transthoracic echocardiography reporting in agreement with recent chamber quantification, diastolic function, and heart valve disease recommendations: an expert consensus document of the European Association of Cardiovascular Imaging. *European heart journal Cardiovascular Imaging*. 2017;18:1301-1310. doi: 10.1093/ehjci/jex244

5. Thygesen K, Alpert JS, Jaffe AS, Chaitman BR, Bax JJ, Morrow DA, White HD. Fourth Universal Definition of Myocardial Infarction (2018). *Circulation*. 2018;138:e618-e651. doi: 10.1161/cir.0000000000000617
