# Supplementary material for: Kinetics and prognostic value of heparin binding protein at the ST-segment-elevation myocardial infarction
Source: Ann Med. 2026 Jan 30;58(1):2622182. doi: 10.1080/07853890.2026.2622182 (PMC12862846; doi:10.1080/07853890.2026.2622182)
Supplement: 2_Clean 2_Tables.docx [file IANN_A_2622182_SM7504.docx]

**Table 1. Clinical characteristics of patients at baseline**

|  | Total  (n = 215) | non-MACE  (n = 169) | MACE  (n = 46) | *P* value |  |
| --- | --- | --- | --- | --- | --- |
| **Demographic and risk factors** |  |  |  |  |  |
| Age, year | 64.17±12.26 | 63.05±12.14 | 68.28±11.95 | 0.011 |  |
| Male, n (%) | 170 (79.07) | 136 (80.47) | 34 (73.91) | 0.332 |  |
| Current smoking, n (%) | 51 (23.72) | 40 (23.67) | 11 (23.91) | 0.517 |  |
| BMI, kg/m^2^ | 24.95±3.65 | 25.07±3.61 | 24.49±3.79 | 0.361 |  |
| Admission heart rate, bpm | 82 (74, 94) | 82 (74, 92) | 84 (77, 105) | 0.036 |  |
| Admission SBP, mmHg | 126 (109, 144) | 127 (110, 146) | 114 (102, 143) | 0.046 |  |
| Admission DBP, mmHg | 77 (67, 87) | 78 (69, 88) | 74 (66, 81) | 0.067 |  |
| **Comorbidities** |  |  |  |  |  |
| Diabetes mellitus, n (%) | 60 (27.91) | 42 (24.85) | 18 (39.13) | 0.056 |  |
| Hypertension, n (%) | 147 (68.37) | 115 (68.05) | 32 (69.57) | 0.844 |  |
| Chronic kidney disease, n (%) | 15 (6.98) | 8 (4.73) | 7 (15.22) | 0.014 |  |
| **Killip classification, n (%)** |  |  |  | <0.001 |  |
| I | 186 (86.5) | 159 (94.1) | 27 (58.7) |  |  |
| II | 16 (7.4) | 11 (6.5) | 5 (10.9) |  |  |
| III-IV | 13 (6.0) | 6 (3.6) | 7 (15.2) |  |  |
| **Culprit lesion, n (%)** |  |  |  | 0.675 |  |
| LAD | 105 (48.8) | 79 (46.7) | 26 (56.5) |  |  |
| LCX | 43 (20.0) | 37 (21.9) | 6 (13.0) |  |  |
| RCA | 67 (31.2) | 52 (30.8) | 15 (32.6) |  |  |
| Stent length, mm | 26.63±6.32 | 26.32±6.43 | 27.97±5.70 | 0.208 |  |
| Stent diameter, mm^2^ | 3.00±0.39 | 3.01±0.40 | 2.97±0.31 | 0.544 |  |
| Non-culprit lesions, n (%) | 146 (67.9) | 117 (69.2) | 29 (63.0) | 0.426 |  |
| **Laboratory values** |  |  |  |  |  |
| WBC, ×10^9^/L | 10.60 (8.76, 12.86) | 10.25 (8.75, 12.19) | 12.22 (8.83, 15.68) | 0.004 |  |
| Hemoglobin, g/L | 138 (125, 150) | 139 (130, 150) | 126 (111, 146) | 0.003 |  |
| Platelet, ×10^9^/L | 214 (177, 254) | 212 (175, 248) | 224 (187, 270) | 0.081 |  |
| Fasting glucose, mmol/L | 6.65 (5.70, 8.74) | 6.59 (5.66, 7.84) | 8.32 (6.19, 12.79) | 0.006 |  |
| HbA1c, % | 6.00 (5.60, 7.00) | 5.90 (5.60, 6.80) | 6.00 (5.70, 8.10) | 0.145 |  |
| Creatine, μmol/L | 78 (67, 96) | 76 (66, 88) | 91 (73, 119) | 0.018 |  |
| eGFR, mL/minute/1.73m^2^ | 84.80 (67.90, 96.50) | 88.10 (73.80, 98.70) | 69.30 (57.00, 80.20) | <0.001 |  |
| Triglyceride, mmol/L | 1.62 (1.13, 2.26) | 1.52 (1.05, 2.01) | 1.71 (1.15, 2.33) | 0.152 |  |
| Total cholesterol, mmol/L | 4.93 (4.05, 5.64) | 4.52 (3.62, 5.48) | 4.95 (4.29, 5.70) | 0.043 |  |
| HDL-C, mmol/L | 1.03 (0.89, 1.22) | 1.03 (0.88, 1.21) | 1.03 (0.91, 1.22) | 0.690 |  |
| LDL-C, mmol/L | 3.03 (2.51, 3.64) | 2.74 (1.95, 3.50) | 3.08 (2.53, 3.69) | 0.048 |  |
| Lp(a), mmol/L | 0.15 (0.07, 0.37) | 0.13 (0.07, 0.32) | 0.31 (0.11, 0.48) | 0.003 |  |
| Peak NT-proBNP, pg/ml | | 1191.00 (497.70, 3115.00) | 824.50 (445.20, 2012.00) | 3648.00 (1560.00, 7502.00) | 0.001 |
| Peak hs-CRP, mg/L | 70.77 (22.07, 160.15) | 43.02 (17.45, 140.17) | 153.22 (107.40, 228.53) | <0.001 |  |
| Peak CK, IU/L | 1084.0 (436.00, 1844.0) | 989.00 (442.00, 1745.00) | 1594.0 (436.00, 2469.00) | 0.191 |  |
| Peak CK-MB, ng/mL | 64.20 (17.50, 130.80) | 59.50 (24.00, 128.80) | 105.95 (10.20, 144.80) | 0.655 |  |
| Peak hs-cTnI, pg/mL | 25989.0 (9588.3, 54320.0) | 24202.0 (9536.1, 50494.0) | 44681.0 (10483.0, 80825.0) | 0.047 |  |
| **Medication** |  |  |  |  |  |
| DAPT, n (%) | 215 (100) | 169 (100) | 46 (100) | 1.000 |  |
| ACEIs/ARBs/ARNI, n (%) | 161 (74.88) | 129 (76.33) | 32 (69.57) | 0.358 |  |
| β-blocker, n (%) | 153 (71.16) | 117 (69.23) | 36 (78.26) | 0.236 |  |
| Statins, n (%) | 200 (93.02) | 160 (94.67) | 40 (86.96) | 0.071 |  |
| MRAs, n (%) | 24 (11.16) | 11 (6.51) | 13 (28.26) | <0.001 |  |
| **Echocardiography** |  |  |  |  |  |
| LA, mm | 38.92±4.24 | 38.60±3.99 | 40.12±4.90 | 0.03 |  |
| LVEDD, mm | 49.86±4.96 | 49.55±4.65 | 50.98±5.90 | 0.03 |  |
| LVESD, mm | 50.57±5.24 | 50.18±4.96 | 52.00±6.01 | <0.01 |  |
| LVEDV, mL | 120.34±28.27 | 118.49±26.33 | 127.14±33.92 | 0.03 |  |
| LVESV, mL | 48 (39, 61) | 45 (38, 56) | 67 (43, 77) | <0.01 |  |
| LVEF, % | 57.26±8.37 | 58.70±7.42 | 51.98±9.57 | <0.01 |  |

Values are mean ± SD, n (%), or median (first quartile, third quartile). Abbreviations: MACE, major adverse cardiac events; BMI, body mass index; HR, heart rate; SBP, systolic blood pressure; DBP, diastolic blood pressure; LAD, left anterior descending; LCX, left circumflex coronary; RCA, right coronary artery; WBC, white blood cell; HbA1c, glycated hemoglobin; eGFR, estimated glomerular filtration; NT-proBNP, N-terminal pro-brain natriuretic peptide; LDL-C, low-density lipoprotein cholesterol; HDL-C, high-density lipoprotein cholesterol; Lp(a), lipoprotein(a); hs-CRP, high sensitivity C reactive protein; CK, creatine kinase; CK-MB, creatine kinase-MB isoenzyme; hs-cTnI, high sensitivity cardiac troponin I; DAPT, dual anti platelet therapy; ACEIs, angiotensin converting enzyme inhibitors; ARBs, angiotensin receptor blockers; ARNI, angiotensin receptor/neprilysin inhibitor; MRAs, mineralocorticoid receptor antagonists; LA, left atrial diameter; LVEDD, left ventricular end-diastolic diameter; LVESD, left ventricular end-systolic diameter; LVEDV, left ventricular end-diastolic volume; LVESV, left ventricular end-systolic volume; LVEF, left ventricular ejection fraction.

**Table 2. Spearman correlations between HBP and other biomarkers at matched time points.**

| **Biomarkers** | **Admission** | | **24h** | | **48h** | | **72h** | |
| --- | --- | --- | --- | --- | --- | --- | --- | --- |
|  | **r** | ***P*** | **r** | ***P*** | **r** | ***P*** | **r** | ***P*** |
| hs-CRP | 0.07 | 0.839 | 0.20 | 0.160 | 0.20 | 0.060 | 0.55 | <0.001 |
| CK | 0.03 | 0.676 | -0.05 | 0.305 | -0.02 | 0.538 | 0.10 | 0.459 |
| CK-MB | -0.01 | 0.956 | -0.01 | 0.858 | -0.01 | 0.684 | 0.24 | 0.271 |
| hs-cTnI | 0.01 | 0.886 | 0.41 | 0.040 | 0.50 | 0.039 | 0.56 | 0.011 |

Correlations were calculated between HBP and the respective biomarkers measured at the same time point. Data are presented as Spearman’s correlation coefficients (r) and *P* values. Abbreviations: HBP, heparin binding protein; hs-CRP, high sensitivity C reactive protein; CK, creatine kinase; CK-MB, creatine kinase-MB isoenzyme; hs-cTnI, high sensitivity cardiac troponin I.

**Table 3.** **Cox models for heparin-binding protein with MACE in patients with STEMI**

|  | Unadjusted  HR (95% CI) | *P* value | Adjusted for model 1 HR (95% CI) | *P* value | Adjusted for model 2  HR (95% CI) | *P* value |
| --- | --- | --- | --- | --- | --- | --- |
| HBP at admission | | | | | | |
| Continuous Per 1 SD increase | 1.003 (0.999, 1.008) | 0.175 | 1.003 (0.999, 1.008) | 0.186 | 1.001 (0.996, 1.007) | 0.661 |
| Quartile 1 | 1 (reference) |  | 1 (reference) |  | 1 (reference) |  |
| Quartile 2 | 1.131 (0.499, 2.564) | 0.769 | 1.244 (0.544, 2.847) | 0.605 | 0.926 (0.381, 2.250) | 0.865 |
| Quartile 3 | 0.633 (0.245, 1.633) | 0.344 | 0.672 (0.260, 1.739) | 0.413 | 1.158 (0.485, 2.768) | 0.741 |
| Quartile 4 | 1.576 (0.731, 3.397) | 0.246 | 1.548 (0.718, 3.338) | 0.265 | 0.620 (0.227, 1.693) | 0.351 |
| HBP 24h | | | | | | |
| Continuous Per 1 SD increase | 1.003 (0.998, 1.009) | 0.260 | 1.002 (0.996, 1.008) | 0.457 | 1.005 (0.999, 1.012) | 0.128 |
| Quartile 1 | 1 (reference) |  | 1 (reference) |  | 1 (reference) |  |
| Quartile 2 | 0.982 (0.409, 2.360) | 0.968 | 0.887 (0.368, 2.135) | 0.897 | 0.879 (0.351, 2.210) | 0.783 |
| Quartile 3 | 1.388 (0.616, 3.129) | 0.429 | 1.278 (0.561, 2.912) | 0.559 | 0.635 (0.242, 1.668) | 0.357 |
| Quartile 4 | 1.133 (0.489, 2.625) | 0.771 | 1.047 (0.451, 2.430) | 0.916 | 0.996 (0.425, 2.331) | 0.992 |
| HBP 48h | | | | | | |
| Continuous Per 1 SD increase | **1.006 (1.001, 1.011)** | **0.017** | **1.006 (1.001, 1.011)** | **0.010** | **1.008 (1.002, 1.014)** | **0.008** |
| Quartile 1 | 1 (reference) |  | 1 (reference) |  | 1 (reference) |  |
| Quartile 2 | 0.862 (0.333, 2.236) | 0.761 | 0.862 (0.332, 2.239) | 0.760 | 0.738 (0.270, 2.019) | 0.554 |
| Quartile 3 | 0.760 (0.283, 2.040) | 0.586 | 0.698 (0.259, 1.885) | 0.479 | 0.673 (0.241, 1.880) | 0.449 |
| Quartile 4 | **2.861 (1.315, 6.224)** | **0.008** | **3.199 (1.458, 7.020)** | **0.004** | 2.326 (0.984, 5.500) | 0.055 |
| HBP 72h | | | | | | |
| Continuous Per 1 SD increase | **1.010 (1.004, 1.016)** | **0.001** | **1.010 (1.005, 1.016)** | **0.001** | **1.010 (1.002, 1.019)** | **0.017** |
| Quartile 1 | 1 (reference) |  | 1 (reference) |  | 1 (reference) |  |
| Quartile 2 | **4.019 (1.121, 14.417)** | **0.033** | **3.660 (1.016, 13.180)** | **0.047** | 3.404 (0.917, 12.637) | 0.067 |
| Quartile 3 | 3.591 (0.988, 13.053) | 0.052 | 3.227 (0.883, 11.787) | 0.076 | 2.926 (0.768, 11.148) | 0.116 |
| Quartile 4 | **8.536 (2.552, 28.551)** | **0.001** | **8.257 (2.468, 27.632)** | **0.001** | **5.533 (1.476, 19.242)** | **0.011** |

Model 1 was adjusted for age and sex; Model 2 was adjusted for model 1 and body mass index, heart rate, systolic blood pressure, smoking, history of hypertension, history of diabetes mellitus, Killip grade, white blood cells, peak of high-sensitivity-C-reactive protein, estimated glomerular filtration rate and peak of high-sensitivity Troponin I. Bold used to highlight those *P* values < 0.05. Abbreviations: STEMI, ST-segment elevation myocardial infarction; HBP, heparin binding protein; MACE, major adverse cardiac events; HR, hazard ratio; CI, confidence interval.

**Table 4. Improvement of accuracy of risk prediction using combination of conventional parameters and HBP**

|  | C-Statistic | NRI (95%CI) | *P* value |
| --- | --- | --- | --- |
| Peak hs-cTnI | 0.796 | Ref | NA |
| Peak hs-cTnI + HBP_72h_ | 0.853 | 0.578 (0.263, 0.894) | <0.001 |
| Peak hs-CRP | 0.638 | Ref | NA |
| Peak hs-CRP + HBP_72h_ | 0.715 | 0.448 (0.128, 0.768) | 0.006 |
| Peak NT-proBNP | 0.744 | Ref | NA |
| Peak NT-proBNP + HBP_72h_ | 0.749 | 0.483 (0.164, 0.803) | 0.003 |
| LVEF | 0.711 | Ref | NA |
| LVEF + HBP_72h_ | 0.804 | 0.503 (0.184, 0.822) | 0.002 |
| Peak hs-cTnI + Peak hs-CRP + Peak NT-proBNP + LVEF | 0.832 | Ref | NA |
| Peak hs-cTnI + Peak hs-CRP + Peak NT-proBNP + LVEF + HBP_72h_ | 0.873 | 0.626 (0.312, 0.940) | <0.001 |

*P* values NRI are for the difference between old models and new models. Abbreviations: NA, not applicable; Ref, reference; NRI, net reclassification index; hs-cTnI, high sensitivity cardiac troponin I; HBP, heparin binding protein; hs-CRP, high sensitivity C reactive protein; NT-proBNP, N-terminal pro-brain natriuretic peptide; LVEF, left ventricular ejection fraction.
