# Supplementary material for: Kinetics and prognostic value of heparin binding protein at the ST-segment-elevation myocardial infarction
Source: Ann Med. 2026 Jan 30;58(1):2622182. doi: 10.1080/07853890.2026.2622182 (PMC12862846; doi:10.1080/07853890.2026.2622182)
Supplement: 2_Clean 2_Figure.docx [file IANN_A_2622182_SM7503.docx]

**Figure Legends**

**Figure 1. The kinetic profile of HBP, hs-CRP, CK, CK-MB and hs-cTnI during the first 72 hours post coronary angiography.** Shown for levels of: (A) HBP, (B) hs-CRP, (C) CK, (D) CK-MB, and (E) hs-cTnI release kinetics in a cohort of patients with (STEMI). Round points denote the median level of the corresponding biomarker at the time measured; admission, 24, 48, and 72 hours. Solid line represents interquartile range. Abbreviations: STEMI, ST-segment elevation myocardial infarction; HBP, heparin-binding protein; hs-CRP, high-sensitive C-reactive protein; CK, creatinine kinase; CK-MB, creatine kinase myocardial band; hs-cTnI, high-sensitive Troponin I.

**Figure 2. Distribution of HBP levels over time according to clinical outcomes.** (A) Plasma HBP levels according to MACE (all-cause mortality, hospitalization for heart failure, relapse MI, or stroke); (B) plasma HBP levels according to hospitalization for heart failure. Wilcoxon matched-pairs signed rank test was used to compare HBP levels at different time points. Abbreviations: HBP, heparin-binding protein; MACE, major adverse cardiac events; HF, heart failure.

**Figure 3. Kaplan–Meier analysis of MACE-free survival according to quartile groups of 72-hour post-pPCI HBP levels.** (A) Kaplan–Meier curve of free from follow-up MACE in the HBP quartile1-2 and HBP quartile 3-4 groups. (B) Landmark analysis discriminating between events occurring before and after 180 days of follow-up.

**Figure 4. Receiver operating characteristic curve for HBP in major adverse cardiac events.** (A) ROC curve analysis of HBP at 48 hours; (B) ROC curve analysis of HBP at 72 hours. Abbreviations: HBP, heparin-binding protein; ROC, receiver operator characteristic curve; AUC, area under the curve.


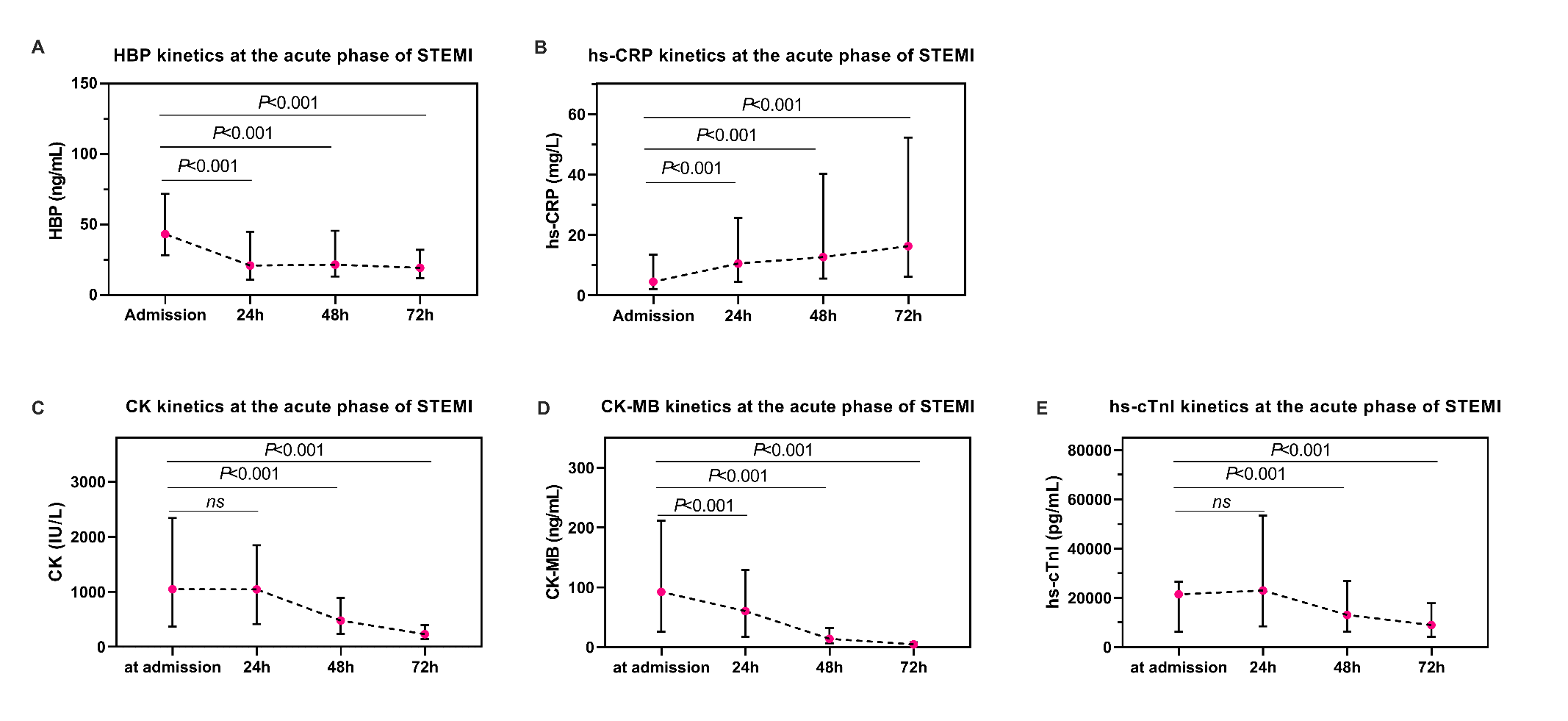
**Figure 1. The kinetic profile of HBP, hs-CRP, CK, CK-MB and hs-cTnI during the first 72 hours post coronary angiography.** Shown for levels of: (A) HBP, (B) hs-CRP, (C) CK, (D) CK-MB, and (E) hs-cTnI release kinetics in a cohort of patients with (STEMI). Round points denote the median level of the corresponding biomarker at the time measured; admission, 24, 48, and 72 hours. Solid line represents interquartile range. Abbreviations: STEMI, ST-segment elevation myocardial infarction; HBP, heparin-binding protein; hs-CRP, high-sensitive C-reactive protein; CK, creatinine kinase; CK-MB, creatine kinase myocardial band; hs-cTnI, high-sensitive Troponin I.


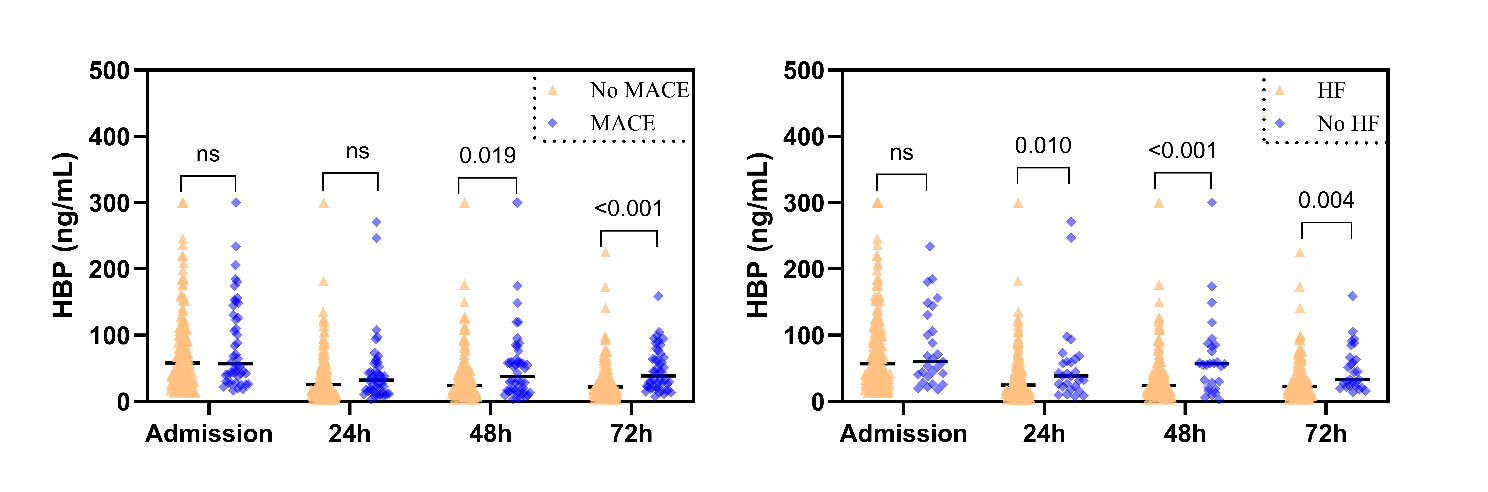
**Figure 2. Distribution of HBP levels over time according to clinical outcomes.** (A) Plasma HBP levels according to MACE (all-cause mortality, hospitalization for heart failure, relapse MI, or stroke); (B) plasma HBP levels according to hospitalization for heart failure. Wilcoxon matched pairs signed rank test was used to compare HBP levels at different time points. Abbreviations: HBP, heparin-binding protein; MACE, major adverse cardiac events; HF, heart failure.


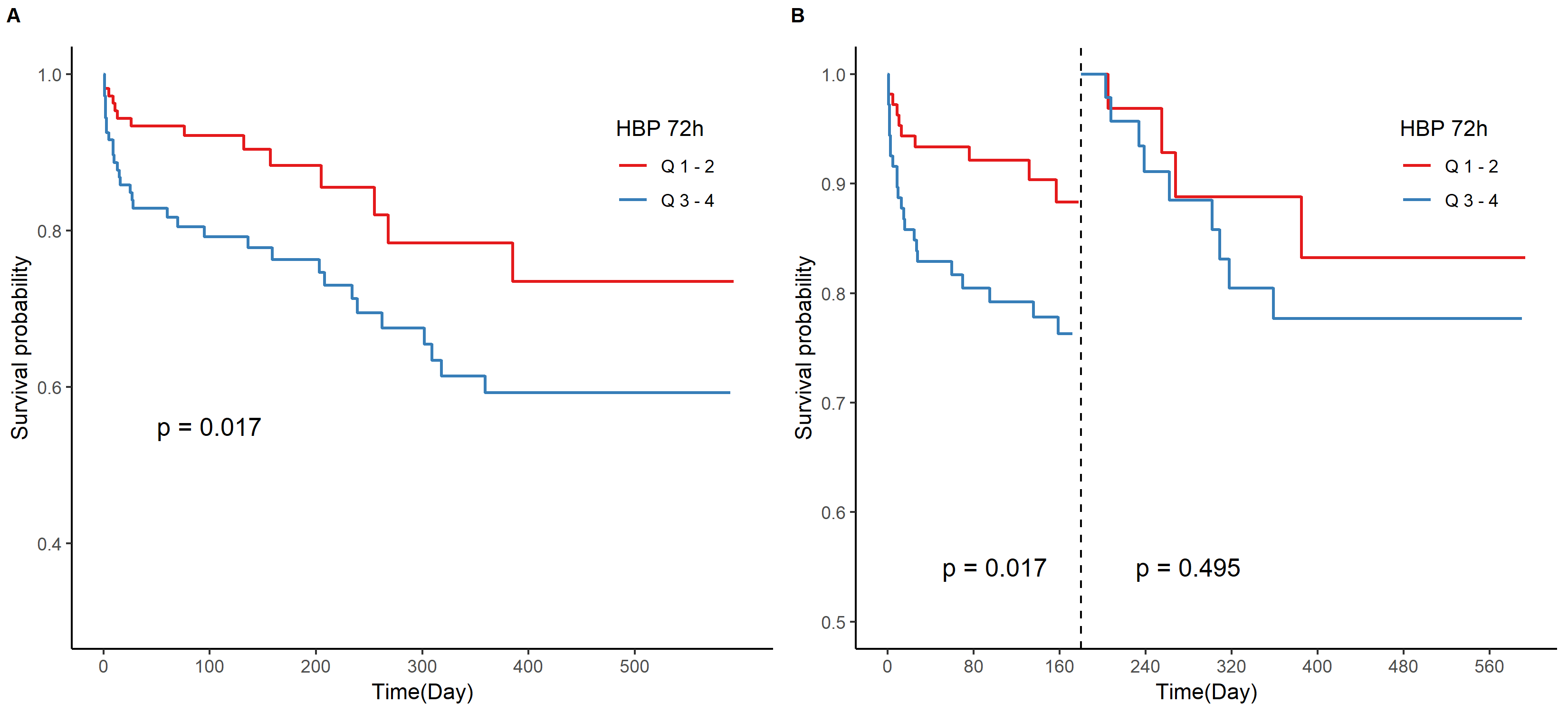
**Figure 3. Kaplan–Meier analysis of MACE-free survival according to quartile groups of 72-hour post-pPCI HBP levels.** (A) Kaplan–Meier curve of free from follow-up MACE in the HBP quartile1-2 and HBP quartile 3-4 groups. (B) Landmark analysis discriminating between events occurring before and after 180 days of follow-up.


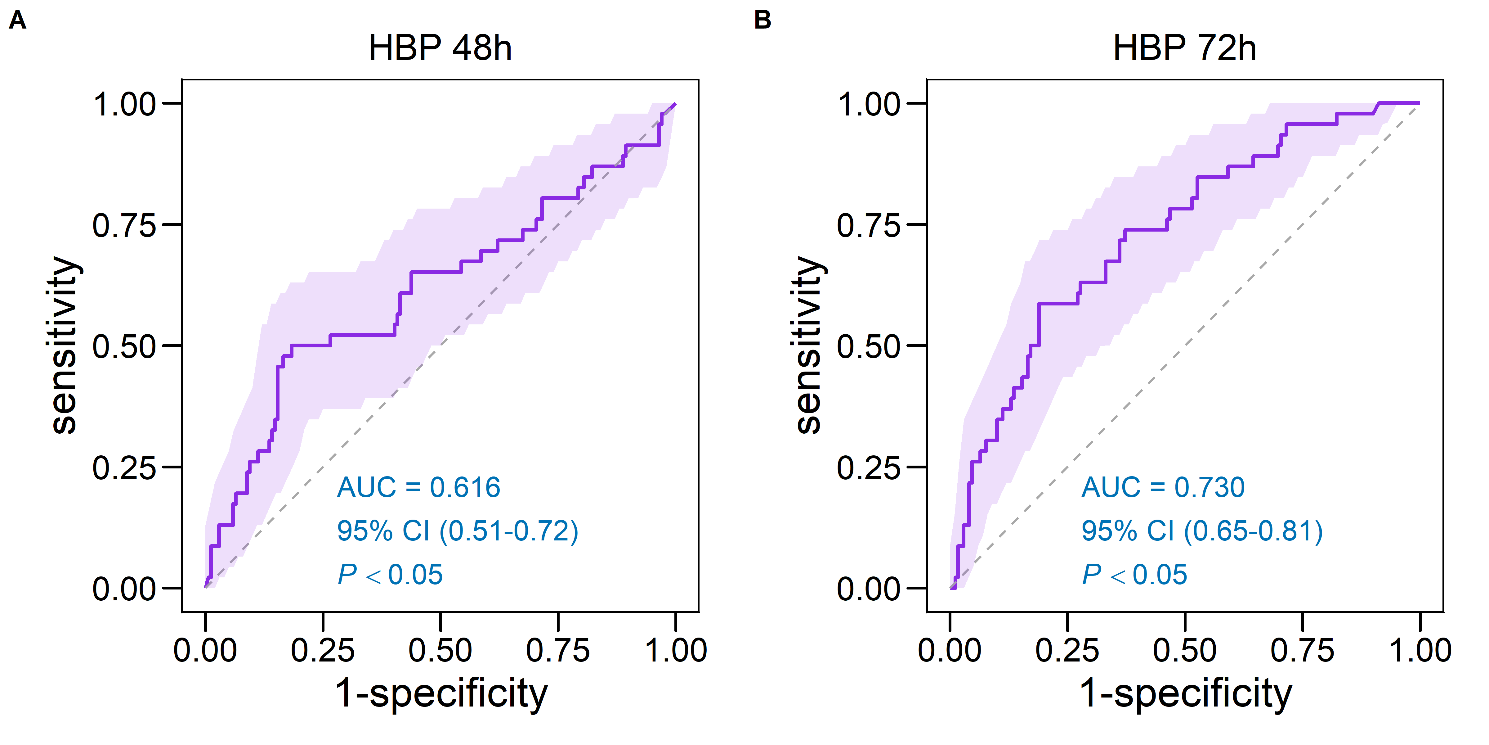
**Figure 4. Receiver operating characteristic curve for HBP in major adverse cardiac events.** (A) ROC curve analysis of HBP at 48 hours; (B) ROC curve analysis of HBP at 72 hours. Abbreviations: HBP, heparin-binding protein; ROC, receiver operator characteristic curve; AUC, area under the curve.
